# High-affinity fluorescent ligands for the 5-HT_3_ receptor

**DOI:** 10.1016/j.bmcl.2011.11.097

**Published:** 2012-01-15

**Authors:** Jonathan Simonin, Sanjeev Kumar V. Vernekar, Andrew J. Thompson, J. Daniel Hothersall, Christopher N. Connolly, Sarah C.R. Lummis, Martin Lochner

**Affiliations:** a Department of Chemistry and Biochemistry, University of Bern, Freiestrasse 3, 3012 Bern, Switzerland; b Center for Drug Design, University of Minnesota, Minneapolis, MN 55455, USA; c Department of Biochemistry, University of Cambridge, Cambridge CB2 1QW, UK; d Centre for Neuroscience, Medical Research Institute, Ninewells Medical School, University of Dundee, Dundee DD1 9SY, UK

**Keywords:** 5-HT_3_R, 5-HT_3_ receptor, nACh, nicotinic acetylcholine, BODIPY, 4,4-difluoro-4-bora-3a,4a-diaza-s-indacene, FITC, fluorescein isothiocyanate, GABA_A_, γ-aminobutyric acid type A, GFP, green fluorescent protein, PEG, polyethylene glycol, SAR, Structure–activity relationship, 5-HT_3_ receptor, Cys-loop ligand-gated ion channels, Fluorescent ligands, Radioligand binding, In vivo imaging

## Abstract

The synthesis, photophysical and biological characterization of a small library of fluorescent 5-HT_3_ receptor ligands is described. Several of these novel granisetron conjugates have high quantum yields and show high affinity for the human 5-HT_3_AR.

## Graphical abstract

**Figure:**
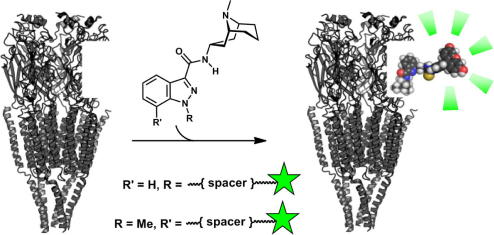

Fluorescence is a useful tool in cell biological studies, and fluorescent labeling of target proteins have enabled numerous in vivo studies of protein function.^1^ Whilst it has become common practice to genetically fuse a fluorescent protein, such as GFP, to a protein of interest, the large size of such fluorescent proteins (e.g., GFP: 238 aa, 27 kDa) can affect the structure and function of the target protein.^2^ Ion channels and transmembrane receptors typically contain several α-helices which are connected via short peptide loops and, given that these proteins undergo large conformational changes, they offer few fusion sites for large fluorescent proteins. An alternative to fusion is to use a low-molecular weight ligand conjugated to a fluorophore. High-affinity fluorescent ligands can be used to visualize the receptor of interest in cells, and also have potential as tracer compounds in fluorescence polarization, and flow cytometry applications that target specific receptors and ion channels.^3^

5-HT_3_Rs are members of the Cys-loop family of ligand-gated ion channels which also includes nACh, GABA_A_, and glycine receptors.^4^ These transmembrane proteins enable rapid synaptic transmission in the central and peripheral nervous system and are composed of five pseudosymmetrically arranged subunits surrounding a central ion-conducting pore. The neurotransmitter binding sites are located in the extracellular N-terminal domains at the interface of two adjacent subunits. Five 5-HT_3_R subunits have been discovered to date (5-HT3A–5-HT3E)^5^ which led to the conclusion that 5-HT_3_R populations most likely comprise several subtypes characterized by distinct functional properties; thus human 5-HT_3_ signaling is more complex than originally anticipated. To date, only homomeric 5-HT_3_A and heteromeric 5-HT_3_AB receptors have been extensively characterized in heterologous systems.^6^ Recent mutagenesis and cysteine modification studies indicate that agonists and antagonists bind to an A–A interface both in human homomeric 5-HT_3_A and heteromeric 5-HT_3_AB receptors which is consistent with their observed identical competitive pharmacologies.^7,8^ Nonetheless, the discovery of subtype-selective molecular tools for the study of 5-HT_3_R populations in native cells and tissue remains an important goal.

Antagonists of 5-HT_3_Rs are used in the clinic to prevent chemotherapy- and radiotherapy-induced nausea and vomiting, post-operative nausea and vomiting, for the treatment of irritable bowel syndrome, and 5-HT_3_R antagonists might be beneficial for the treatment of psychiatric and neurological disorders, such as anxiety, drug dependence and bulimia nervosa.^9,10^ They have also been shown to reduce pain in certain conditions including rheumatoid arthritis, fibromyalgia and migraine.^11^

As a complementary approach to conventional biological methods such as site-directed mutagenesis, radioligand binding and electrophysiology, we are developing biophysical small-molecular probes to investigate the structure and function of ligand-gated ion channels. Previously, we undertook a SAR study of the high-affinity competitive 5-HT_3_R antagonist granisetron^12^ (**1**, Fig. 1, *K*_i_ = 1.45 nM), and identified positions on the granisetron core which were tolerant to substitution.^13^ In this initial study we discovered that methoxy-substituted granisetrons **2**–**5** are quite fluorescent and some of them bound with high affinity to the 5-HT_3_R. However, their quantum yields were poor (Table 1). We subsequently conjugated a commercial fluorophore, BODIPY FL, to the N1-position of granisetron and obtained high-affinity probe **6**, which had much higher fluorescence intensity, and was used to visualize recombinant 5-HT_3_ARs in mammalian cells.^13^ Unfortunately, **6** gave high fluorescence background in gut preparations and primary neurons that could not be washed out. These limitations prompted us to generate a small library of fluorescent 5-HT_3_R ligands with improved properties. Herein, we describe the synthesis, photophysical and biological characterization of these novel granisetron–fluorophore conjugates.

The synthesis of the N1-conjugated compound series is depicted in Schemes 1–3. Amide **7**^13^ was first N-alkylated with either aliphatic aminopropyl-, aminobutyl- or more polar PEG-linker building blocks **8**–**10**. Subsequent Boc-deprotection liberated the primary amino group that was used to couple various fluorophores **F1**–**F5** to the granisetron-linker constructs **11**–**13** (Scheme 2). For the synthesis of probes **26**–**28** it was more advantageous to couple the linker to the fluorophore first, and then perform the N1-alkylation in the second step (Scheme 3). Unexpectedly, when we condensed 4-bromobutanoyl chloride with 2,4-dimethylpyrrole following a literature protocol^14^ we obtained BODIPY dye **25** as the sole product where two moles of acyl chloride have reacted with two moles of the pyrrole. The structure of **25** was confirmed by full spectroscopic characterization and a crystal structure.

Other than the N1-position on granisetron, we identified the C7-position as tolerant to substitution in our initial SAR study.^13^ Therefore we synthesized probes **36**–**39**, where the fluorophores were conjugated to this latter position via different linkers. The synthesis of 7-hydroxy granisetron **31** was described previously; we have, however, found a more practical and scalable route to access this intermediate (Scheme 4). The indazole-3-carboxylate **29** was synthesized using a 1,3-dipolar cycloaddition of in situ generated aryne with a diazo ester.^15^ Only the C7-substituted regioisomer was obtained in this reaction. This was followed by ester hydrolysis, amide formation with bicyclic amine **30**, selective N1-methylation and methyl ether cleavage. It was crucial to follow the above order of steps since N-methylation of the indazole ester **29** also yields small amounts of N2-methylated side product which is extremely difficult to separate from the desired N1-isomer. The hydroxyl group of **31** was alkylated with protected spacers **8**, **10** or **32** and the Boc-group was subsequently cleaved. Finally, 7-(diethylamino)-coumarin-3-carboxylic acid or FITC was coupled to the spacers to yield probes **36**–**39**.

The photophysical properties of fluorescent granisetron derivatives were measured in MeOH and/or phosphate buffer at pH 7 (Table 1). The granisetron probes that have fluorescein (**16**, **18** and **21**) or BODIPY dyes (**6**, **14** and **28**) appended to N1 show the highest quantum yields (*Φ*_f_). The quantum yield of the coumarin-containing probes **15**, **17**, **20**, **36**–**38** was low in pH 7 buffer, but was substantially increased in less polar solvents (e.g., for **15**: *Φ*_f_ (CH_2_Cl_2_) = 0.55). The binding affinities of the fluorescent granisetron probes for the human 5-HT_3_AR were determined by competition binding studies with [^3^H]granisetron. Probes **14**, **16**, **18**, **27**, **28** and **36** exhibited affinities similar to the parent compound granisetron (Table 1) and thus could be useful as tracer ligands for fluorescence-based binding assays and tools for imaging. In terms of probe design it appears that optimal binding is obtained if the fluorophore is conjugated to N1 of granisetron via a short (butyl) aliphatic spacer.

The utility of granisetron probes **16**–**18**, **26**–**28** and **36** to fluorescently label the receptor was studied using live cell imaging of COS-7 cells transiently transfected with human 5-HT_3_A receptors. Only probes **16**, **18** and **28** gave detectable staining. Probes **16** and **18** produced selective staining for 5-HT_3_R as demonstrated by fluorescence at the periphery (plasma membrane) of transfected cells, whilst this fluorescence was absent in mock transfected cells (Fig. 2). Furthermore, 5-HT_3_R staining with these probes was inhibited by co-incubation with the 5-HT_3_R antagonist ondansetron (OND, 10 μM) (Fig. 2). In contrast, probe **28** produced intense fluorescence in both 5-HT_3_A- and mock-transfected cells, and was not blocked by ondansetron (Fig. 2). These data suggest nonspecific interactions of this probe with the cells.

In summary, we have designed and synthesized a small library of fluorescent 5-HT_3_ receptor ligands. Most notably, novel granisetron conjugates **14**, **16**, **18**, **27** and **28** have similar binding affinities for the human 5-HT_3_AR as measured for the parent compound **1**. This is in agreement with previous studies which showed that bulky fluorophores can be appended via short aliphatic linker to the N1-position of granisetron. Moreover, these high affinity probes exhibit high quantum yields and emission maxima above 500 nm in polar media. Probes **16** and **18**, in particular, show specific fluorescent labeling of the human 5-HT_3_AR in live cells. We are currently focusing our efforts on evaluating our probes in fluorescence polarization and flow cytometry applications.

## Figures and Tables

**Figure 1:**
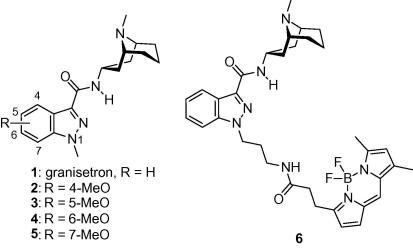
Reference compound and fluorescent granisetron derivatives.

**Figure 2:**
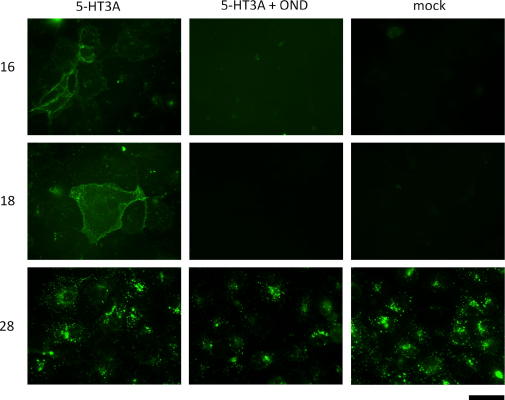
Fluorescent labeling of human 5-HT_3_AR in live COS-7 cells. Cells were either transfected with human 5HT3A cDNA (left and middle panels), or mock transfected (right panels). 24 h later they were incubated with 100 nM of probes **16**, **18** or **28** in HBS buffer for 1 h at room temperature in the dark. The cells were imaged using a fluorescence microscope set to the appropriate absorption/emission wavelengths (Table 1). Some cells were also co-incubated with 10 μM ondansetron (OND, middle panels) to block 5-HT_3_ receptors. Scale bar represents 50 μm.

**Scheme 1:**
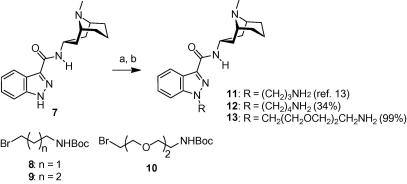
Synthesis of intermediates **11**–**13**. Reagents and conditions: (a) for **11** and **12**: *t*-BuOK, THF/DMF 5:1, 0 °C; **8** or **9**, 0 °C to rt. For **13**: K_2_CO_3_, *n*-Bu_4_NI, **10**, DMF, 60 °C; (b) 4 M HCl in 1,4-dioxane, rt.

**Scheme 2:**
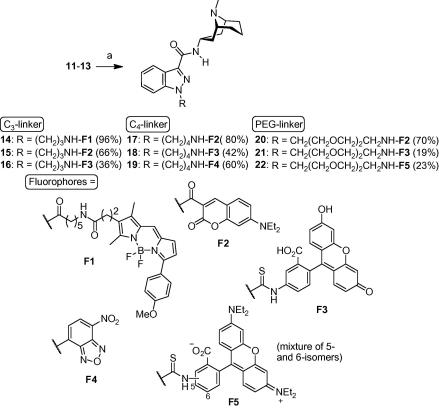
Synthesis of granisetron probes **14**–**22**. Reagents and conditions: (a) for **14**: **11**, *i*-Pr_2_EtN, BODIPY TMR-X succinimidyl ester, DMF, rt. For **15**, **17** and **20**: **11**–**13**, DCC, HOBt, Et_3_N, DMF/CH_2_Cl_2_; 7-(diethylamino)coumarin-3-carboxylic acid, rt. For **16**, **18** and **21**: **11**–**13**, FITC, Et_3_N, DMF, rt. For **19**: **12**, Na_2_CO_3_, NBD–Cl, DMF/THF/H_2_O, 45 °C. For **22**: **13**, rhodamine B 5(6)-isothiocyanate, Et_3_N, DMF, rt.

**Scheme 3:**
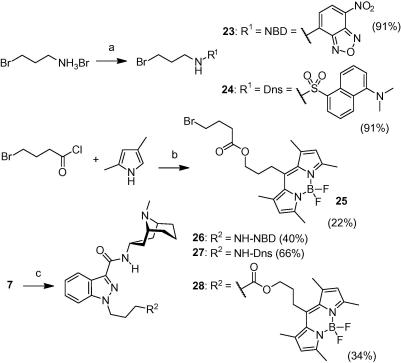
Synthesis of granisetron probes **26**–**28**. Reagents and conditions: (a) for **23**: NBD–Cl, Et_3_N, THF/H_2_O 4:1, rt. For **24**: Dns–Cl, Et_3_N, CH_2_Cl_2_, rt; (b) CH_2_Cl_2_, 50 °C; *i*-Pr_2_EtN, CH_2_Cl_2_/toluene, rt; BF_3_·Et_2_O, 50 °C; (c) *t*-BuOK, THF/DMF 5:1, 0 °C; **23**–**25**, 0 °C to rt.

**Scheme 4:**
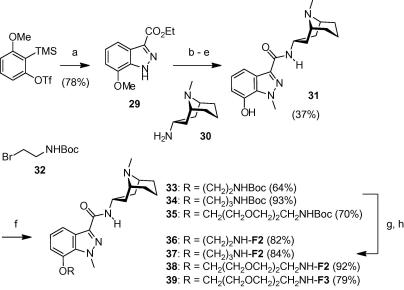
Synthesis of granisetron probes **36**–**39**. Reagents and conditions: (a) N_2_CHCO_2_Et, TBAF, THF, −78 °C to rt; (b) 2 M NaOH, MeOH, rt; (c) DCC, HOBt, DMF/CH_2_Cl_2_; **30**, rt; (d) *t*-BuOK, THF/DMF 4:1, 0 °C; MeI, 0 °C to rt; (e) 1 M BBr_3_ in CH_2_Cl_2_, CH_2_Cl_2_, rt; (f) K_2_CO_3_, *n*-Bu_4_NI, **8**, **10** or **32**, DMF, 60 °C; (g) 4 M HCl in 1,4-dioxane, rt; (h) for **36**–**38**: DCC, HOBt, DMF/CH_2_Cl_2_; 7-(diethylamino)coumarin-3-carboxylic acid, rt. For **39**: FITC, Et_3_N, DMF, rt.

**Table 1:** Photophysical properties of fluorescent granisetron probes and their binding affinities for the human 5-HT_3_AR

Probe	*λ*_max_ Abs (nm)	*ε* (M^−1^ cm^−1^)	*λ*_max_ Em (nm)	*Φ*_f_	*K*_i_ (nM)
					mean ± SEM
**1**	—	—	—	—	1.45 ± 0.13^a^
**2**	298^b^	10,900^b^	388^b^	0.01^b^	26 ± 7^c^
**3**	326^b^	—	383^b^	0.02^b^	5,300 ± 200^c^
**4**	300^b^	—	375^b^	0.01^b^	3,000 ± 1,000^c^
**5**	302^b^	7,800^b^	413^b^	0.03^b^	71 ± 8^c^
**6**	504^b^	89,200^b^	511^b^	0.61^b^	2.8 ± 0.7^c^
**14**	536^b^	8,700^b^	573^b^	0.48^b^	0.9 ± 0.3
**15**	432^d^	37,100^b^	476^d^	0.04^d^	199 ± 39
**16**	495^d^	13,500^b^	518^d^	0.66^d^	1.6 ± 0.3
**17**	432^d^	78,000^b^	471^d^	0.05^d^	7.3 ± 2.5
**18**	493^d^	18,600^b^	519^d^	0.69^d^	1.1 ± 0.2
**19**	465^b^	22,600^b^	536^b^	0.13^b^	NB
**20**	431^d^	71,900^b^ (29,000^d^)	480^d^	0.04^d^	142 ± 5
**21**	498^d^	17,700^b^ (70,700^d^)	524^d^	0.68^d^	6,300 ± 1,600
**22**	557^d^	53,100^d^	584^d^	0.24^d^	384 ± 68
**26**	465^b^	20,400^b^	540^b^	0.10^b^	8.7 ± 5.8
**27**	350^b^	11,500^b^	524^b^	0.29^b^	1.2 ± 0.6
**28**	497^b^	78,800^b^	505^b^	0.44^b^	1.6 ± 0.8
**36**	430^d^	40,100^b^	479^d^	0.02^d^	1.9 ± 0.9
**37**	430^d^	40,600^b^	478^d^	0.05^d^	157 ± 9
**38**	437^d^	21,800^d^	484^d^	0.06^d^	1,300 ± 300
**39**	498^d^	44,200^d^	519^d^	0.19^d^	208 ± 55

a From Ref. 16.

b In MeOH.

c From Ref. 13.

d In phosphate buffer pH 7; —, not attempted; NB, no binding.

## Supplementary data

Supplementary data Synthesis details and spectral data for all compounds, crystal structure of **25**, HPLC purity assessment for target compounds and experimental details for photophysical measurements.
