# Supplementary material for: High-affinity fluorescent ligands for the 5-HT3 receptor
Source: Bioorg Med Chem Lett. 2012 Jan 15;22(2):1151–5. doi: 10.1016/j.bmcl.2011.11.097 (PMC3277886; doi:10.1016/j.bmcl.2011.11.097)

Supporting Information

High-affinity fluorescent ligands for the 5-HT_3 receptor

Jonathan Simonin,[a] Sanjeev Kumar V. Vernekar,[b] Andrew J. Thompson,[c] J. Daniel Hothersall,[d] Christopher Connolly,[d] Sarah C. R. Lummis[c] and Martin Lochner*[a]

[a] *Department of Chemistry and Biochemistry, University of Bern, Freiestrasse 3, 3012 Bern, Switzerland. Fax: +41-31-631-4272; Tel: +41-31-631-3311; E-mail: martin.lochner@dcb.unibe.ch*
[b] *Center for Drug Design, University of Minnesota, 516 Delaware Street S. E., MMC 204, Minneapolis, MN 55455, USA*
[c] *Department of Biochemistry, University of Cambridge, Tennis Court Road, Cambridge CB2 1QW, UK*
[d] *Centre for Neuroscience, Ninewells Medical School, University of Dundee, Dundee DD1 9SY, UK*

TABLE OF CONTENTS

(1) Materials and methods for synthesis of the compounds S2
(2) Synthesis of *N*1-linked granisetron conjugates S2
(3) Synthesis of C7-linked granisetron conjugates S16
(4) Fluorescence spectroscopy measurements S22
(5) Radioligand binding and fluorescence microscopy S23
(6) X-Ray structure of **25** and ^{1}H-NMR spectra of the final compounds S24

(1) Materials and methods

Reactions were monitored by thin layer chromatography (TLC) on silica gel ALUGRAM Xtra SIL G/UV$_{254}$ plates. Visualization was performed under UV (254 nm) or by staining with a solution of $KMnO_4$ and subsequent heating. Purification of crude products was performed by flash chromatography on silica gel (0.040-0.63 mm, Fluka Analytical 60752 silica gel 60, 230-400 mesh) or on aluminum oxide 5016-A-I Basic from CAMAG and then monitored with POLYGRAM ALOX N/UV$_{254}$ plates from Macherey-Nagel.

^{1}H-NMR and ^{13}C spectra were recorded at 300 and 400 MHz and 75 and 100 MHz respectively, on a Bruker Avance 300 and DPX-400. Chemical shifts are reported in δ (ppm) and the residual signal of the solvent was used as the internal standard ($CDCl_3$ ^{1}H: δ = 7.26 ppm, ^{13}C: δ = 77.0 ppm, CD_3OD ^{1}H: δ = 3.31 ppm, ^{13}C: δ = 49.1 ppm, D_2O ^{1}H: δ = 4.79 ppm). HSQC spectra were recorded on a Bruker Avance 400 spectrometer. High resolution mass spectra were obtained using Electro spray ionization mass (MS-ESI) technique on a Applied Biosystems Sciex QSTAR Pulsar (hybrid quadrupole time-of-flight mass spectrometer) instrument. Infrared spectra (IR) were recorded on a *Jasco* FT-IR-460 Plus spectrometer equipped with a Specac MKII Golden Gate Single Reflection Diamond ATR System neat or as film with CH_2Cl_2. Reverse phase purification was done with preparative-LC, Waters PrepLC System and UV detection at 214 nm. Purity of the compounds was determined by UPLC on a *Dionex* Ultimate 3000 using a C8 or a C18 reversed-phase column, the gradient used were 100% A to 100% D over 5 min. or 70% A 30% D to 100% D over 6 min (A = 100% water with 0.1% of trifluoroacetic acid and D = 40% water with 60% acetonitrile and 0.1% trifluoroacetic acid). Purity of some compounds was also determined by reversed-phase HPLC (Zorbax Eclipse XDB-C18, 5μm, 4.6 × 150 mm) using a MeOH/H_2O solvent system. Compounds were eluted with a gradient of 35% MeOH/H_2O to 100% MeOH with 1% NH_4OH for 55 min at a flow rate of 1 ml/min. Purity was determined by total absorbance at 254 nm. Lyophilizations were performed with a *Christ* Alpha 2-4 LD plus device.

Solvents and reagents were purchased from Sigma, Aldrich, Fluka, Acros and Alfa Aesar and used without further purification. Anhydrous solvents were used preferentially for reactions. Reactions were always performed under inert atmosphere (N_2).

(2) Synthesis of *N*1-linked granisetron conjugates

N-(3-bromopropyl)-7-nitrobenzo[*c*][1,2,5]oxadiazol-4-amine (23)

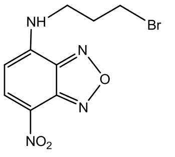

$C_9H_9BrN_4O_3$
Exact Mass: 299.9858

To a solution of 3-Bromopropylamine hydrobromide (0.2 g, 0.92 mmol) in THF/H_2O (4:1, 10 ml) was added Et_3N (0.27 g, 2.7 mmol), followed by NBD-Cl (0.27 g, 1.4 mmol), the mixture was then stirred at room temperature. The reaction was carried out in the absence of light. The progress of the reaction was monitored by TLC and completed in 24 h. The solvents were removed *in vacuo* and the reaction mixture was extracted with EtOAc (3×20 ml) and washed with water. The combined organic phases were dried over Na_2SO_4, filtered and concentrated to give the crude product. The crude product was further purified by flash column chromatography (EtOAc/hexane; 1.5:8.5) to afford **23** (0.25 g, 0.83 mmol, 91%) as a yellow solid: mp 78-80°C. R_f = 0.04 (EtOAc/hexane; 2:8). IR (neat): 3369, 1578,

1493, 1289, 1245, 1113, 971, 830, 737 cm^{-1}. ^{1}H-NMR ($CDCl_3$, 400 MHz): δ 8.51 (d, *J* = 8.57 Hz, 1H) ; 6.33 (br, 1H) ; 6.28 (d, *J* = 8.57 Hz, 1H) ; 3.78-3.73 (m, 2H) ; 3.57 (t, *J* = 6.03 Hz, 2H) ; 2.40-2.34 (m, 2H). ^{13}C-NMR ($CDCl_3$, 100 MHz): δ 144.3 ; 143.6 ; 136.3 ; 98.9 ; 42.2 ; 30.9 ; 29.8. HRMS-ESI (+) *m/z* calcd for $C_9H_9BrN_4O_3Na$ 322.9756 $[M+Na]^+$, found 322.9750 $[M+Na]^+$; Anal.calcd C 35.90%, H 3.01%, N 18.61%, found C 36.51%, H 3.10%, N 18.37%.

N-((*endo*)-9-methyl-9-azabicyclo[3.3.1]nonan-3-yl)-1-(3-((7-nitrobenzo[*c*][1,2,5] oxadiazol-4-yl)amino)propyl)-1*H*-indazole-3-carboxamide (26)

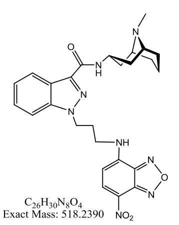

Amide **7**[13] (0.06 g, 0.20 mmol) was dissolved in dry DMF/THF (1:5, 6ml), cooled to 0°C and stirred for 5 min. Then a solution of *t*-BuOK (0.049 g, 0.4 mmol) in dry THF (1 ml) was added drop-wise at 0°C and stirred for 15 min, followed by a solution of compound **23** (0.078 g, 0.26 mmol) in dry THF (1 ml), the mixture was warmed to room temperature and stirred for 12h. The reaction was carried out in the absence of light and under N_2 atmosphere. The progress of the reaction was monitored by TLC. The solvents were removed *in vacuo* and the crude product was purified by flash column chromatography (CH_2Cl_2 then CH_2Cl_2/MeOH/Et_3N; 96:3:1) to afford **26** (0.04 g, 0.077 mmol, 40%) as a brown solid: R_f = 0.16 (MeOH/CH_2Cl_2; 3:7). IR (neat): 2926, 1643, 1529, 1291, 1250, 1175, 807, 739 cm^{-1}. ^{1}H-NMR (CD_3OD, 400 MHz): δ 8.26 (d, *J* = 8.82 Hz, 1H) ; 8.09 (d, *J* = 8.20 Hz, 1H) ; 7.55 (d, *J* = 8.56 Hz, 1H) ; 7.33 (t, *J* = 7.60 Hz, 1H) ; 7.16 (t, *J* = 7.53 Hz, 1H) ; 6.04 (d, *J* = 8.82 Hz, 1H) ; 4.62 (t, *J* = 6.34 Hz, 2H) ; 4.58 (tt, *J* = 6.9 Hz, *J* = 11.6 Hz, 1H) ; 3.56 (br, 2H) ; 3.07 (d, *J* = 10.36 Hz, 2H) ; 2.50 (s, 3H) ; 2.48-2.37 (m, 4H) ; 2.08-1.90 (m, 3H) ; 1.54-1.45 (m, 3H) ; 1.10-1.07 (m, 2H). ^{13}C-NMR (CD_3OD, 100 MHz): δ 164.0 ; 142.4 ; 138.7 ; 137.5 ; 136.3 ; 128.1 ; 124.0 ; 123.7 ; 123.2 ; 110.9 ; 99.7 ; 52.9 ; 47.7 ; 41.5 ; 40.6 ; 33.4 ; 29.6 ; 25.6 ; 14.9. HRMS-ESI (+) m/z calcd for $C_{26}H_{31}N_8O_4$ 519.2468 $[M+H]^+$, found 519.2463 $[M+H]^+$. UV-Vis/Fluo (MeOH): $\lambda_{max\ abs}$ 465 nm, $\lambda_{max\ em}$ 540 nm. Purity HPLC 96%, t_R = 17.31 min.

N-(3-bromopropyl)-5-(dimethylamino)naphthalene-1-sulfonamide (24)

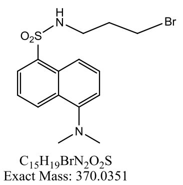

To a solution of 3-Bromopropylamine hydrobromide (0.19 g, 0.88 mmol) in CH_2Cl_2 (10 ml) was added Et_3N (0.27 g, 2.7 mmol) followed by dansylchloride (0.2 g, 0.74 mmol), the mixture was stirred at room temperature. The reaction was carried out in the absence of light. The progress of the reaction was monitored by TLC and completed in 12 h. The reaction mixture was diluted with CH_2Cl_2 (10 ml) and washed with aqueous Na_2CO_3 (5 ml). The CH_2Cl_2-layer was dried over Na_2SO_4, filtered and concentrated to give the crude product. The crude product was further purified by flash column chromatography (EtOAc/hexane; 1:9) to afford **24** (0.25 g, 0.68 mmol, 91%) as a green oil: R_f = 0.08 (EtOAc/hexane; 2:8). IR (neat): 3286, 2937, 2830, 1570, 1309, 1138, 1061, 786 cm^{-1}. ^{1}H-NMR ($CDCl_3$, 400 MHz): δ 8.56 (d, *J* = 8.50 Hz, 1H) ; 8.29-8.25 (m, 2H) ; 7.59-7.51 (m, 2H) ; 7.19 (d, *J* = 7.54 Hz, 1H) ; 4.86 (br, 1H) ; 3.31 (t, *J* = 6.27 Hz, 2H) ; 3.06 (q, *J* = 6.43 Hz, 2H) ; 2.89 (s, 6H) ; 1.97-1.91 (m, 2H). ^{13}C-NMR ($CDCl_3$, 100 MHz): δ 152.2 ; 134.4 ; 130.7 ; 130.0 ; 129.9 ; 129.6 ; 128.6 ; 123.2 ; 118.5 ; 115.3 ; 45.4 ; 41.5 ; 32.3 ; 30.2. HRMS-ESI (+) *m/z* calcd for $C_{15}H_{20}BrSN_2O_2$ 371.0429 $[M+H]^+$, found 371.0423 $[M+H]^+$; Anal.calcd C 48.52%, H 5.16%, N 7.54%, found C 48.37%, H 5.08%, N 7.63%.

1-(3-(5-(dimethylamino)naphthalene-1-sulfonamido)propyl)-*N*-((*endo*)-9-methyl-9-azabicyclo[3.3.1]nonan-3-yl)-1*H*-indazole-3-carboxamide (27)

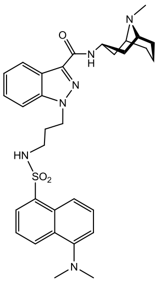

$C_{32}H_{40}N_6O_3S$
Exact Mass: 588.2883

Amide **7** (0.1 g, 0.34 mmol) was dissolved in dry DMF/THF (1:5, 5 mL), cooled to 0°C and stirred for 5 min. Then a solution of *t*-BuOK (0.053 g, 0.44 mmol) in dry THF (1 ml) was added drop-wise at 0°C and stirred for 15 min, followed by a solution of compound **24** (0.14 g, 0.37 mmol) in dry THF (1 ml), the mixture was warmed to room temperature and stirred for 12h. The reaction was carried out in the absence of light and under N_2 atmosphere. The progress of the reaction was monitored by TLC. The solvents were removed *in vacuo* and the crude product was purified by flash column chromatography (CH_2Cl_2 then CH_2Cl_2/MeOH/Et_3N; 96:3:1) to afford **27** (0.13 g, 0.22 mmol, 66%) as a green solid: R_f = 0.2 (MeOH/CH_2Cl_2; 3:7). IR (neat): 2920, 2374, 1639, 1533, 1308, 788, 750 cm^{-1}. ^{1}H-NMR (CD_3OD, 400 MHz): δ 8.48 (d, *J* = 8.52 Hz, 1H) ; 8.34 (d, *J* = 8.66 Hz, 1H) ; 8.17 (d, *J* = 8.18 Hz, 1H) ; 8.06 (d, *J* = 7.21 Hz, 1H) ; 7.54 (t, *J* = 8.12 Hz, 1H) ; 7.46 (t, *J* = 7.93 Hz, 1H) ; 7.34- 7.31 (m, 2H) ; 7.23-7.20 (m, 2H) ; 4.54 (tt, *J* = 6.8 Hz, *J* = 11.2 Hz, 1H) ; 4.34 (t, *J* = 6.68 Hz, 2H) ; 3.09 (d, *J* = 10.60 Hz, 2H) ; 2.89 (t, *J* = 6.62 Hz, 2H) ; 2.82 (s, 6H) ; 2.50 (s, 3H) ; 2.46-2.38 (m, 2H) ; 2.08-1.92 (m, 5H) ; 1.59-1.44 (m, 3H) ; 1.12-1.08 (m, 2H). ^{13}C-NMR (CD_3OD, 100 MHz): δ 161.6 ; 156.7 ; 153.3 ; 142.2 ; 138.4 ; 137.4 ; 131.3 ; 130.9 ; 130.2 ; 129.2 ; 124.3 ; 123.9 ; 123.7 ; 123.1 ; 120.4 ; 116.4 ; 110.8 ; 52.9 ; 47.2 ; 45.8 ; 41.4 ; 41.3 ; 40.6 ; 33.3 ; 30.6 ; 25.7 ; 14.9. HRMS-ESI (+) *m/z* calcd for $C_{32}H_{41}SN_6O_3$ 589.2961 $[M+H]^+$, found 589.2955 $[M+H]^+$. UV-Vis/Fluo (MeOH): $\lambda_{max\ abs}$ 350 nm, $\lambda_{max\ em}$ 524 nm. Purity HPLC 99%, t_R = 25.33 min.

10-(3-((4-Bromobutanoyl)oxy)propyl)-5,5-difluoro-1,3,7,9-tetramethyl-5*H* dipyrrolo[1,2-*c*:2',1'-*f*][1,3,2]diazaborinin-4-ium-5-uide (25)

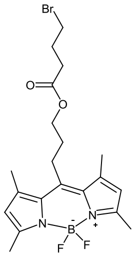

$C_{20}H_{26}BBrF_2N_2O_2$
Exact Mass: 454.12

4-bromobutanoyl chloride (1.52 g, 16.0 mmol) was added drop-wise to a stirred solution of 2,4-dimethylpyrrole (2.22 g, 12.0 mmol) in CH_2Cl_2 (60 ml) and the mixture was heated at 50°C for 2 h. After evaporation of the solvent, toluene (150ml), CH_2Cl_2 (10 ml) and DIEA (9.2 g, 0.072) were added to the solid and the mixture was stirred at room temperature for 30 min. followed by addition of BF_3•Et_2O (16.9 g, 120 mmol). The reaction mixture was heated at 50°C for 1.5 h. The solvents were removed *in vacuo* and the residue was extracted with CH_2Cl_2 (3×30 ml) and washed with 2M NaOH. The combined organic phases were dried over Na_2SO_4, filtered and concentrated to give the crude product. The crude product was further purified by flash column chromatography (EtOAc/hexane; 1:9) to afford **25** (0.8 g, 1.76 mmol, 22%) as an orange solid: mp 114-116°C. R_f = 0.28 (EtOAc/Hexane; 1:4). IR (neat): 2925, 1581, 1531, 1491, 1293, 1174, 1038, 997, 750 cm^{-1}. ^{1}H-NMR ($CDCl_3$, 300 MHz): δ 6.04 (s, 2H) ; 4.22 (t, *J* = 6.13 Hz, 2H) ; 3.45 (t, *J* = 6.35 Hz, 2H) ; 3.04-2.98 (m, 2H) ; 2.53-2.49 (m, 8H) ; 2.41 (s, 6H) ; 2.16 (dd, *J* = 6.77 Hz, *J* = 6.94 Hz, 2H) ; 1.99 -1.92 (m, 2H). ^{13}C-NMR ($CDCl_3$, 175 MHz): δ 171.9 ; 153.7 ; 144.1 ; 139.6 ; 130.7 ; 121.2 ; 63.5 ; 32.1 ; 31.6 ; 30.0 ; 27.0 ; 24.5 ; 15.0 ; 13.9. HRMS-ESI (+) *m/z* calcd for $C_{20}H_{27}BBrF_2N_2O_2$ 477.1136 $[M+H]^+$, found 477.1134 $[M+H]^+$; Anal.calcd C 52.78%, H 5.76%, N 6.15%, found C 53.29%, H 5.77%, N 6.18%. Crystal structure *vide infra*.

5,5-difluoro-1,3,7,9-tetramethyl-10-(3-((4-(3-(((*endo*)-9-methyl-9azabicyclo[3.3.1] nonan-3-yl)carbamoyl)-1*H*-indazol-1-yl)butanoyl)oxy)propyl)-5*H*-dipyrrolo[1,2-*c*:2',1'-*f*][1,3,2]diazaborinin-4-ium-5-uide (28)

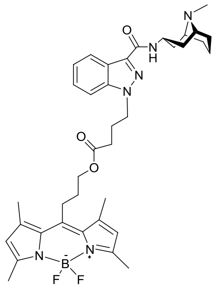

$C_{37}H_{47}BF_2N_6O_3$
Exact Mass: 672.3771

Amide **7** (0.14 g, 0.47 mmol) was dissolved in dry DMF/THF (1:5, 8 ml), cooled to 0°C and stirred for 5 min. Then a solution of *t*-BuOK (0.063 g, 0.52 mmol) in dry THF (1 ml) was added drop-wise at 0°C and stirred for 15 min, followed by a solution of compound **25** (0.32 g, 0.7 mmol) in dry THF (2 mL). The mixture was warmed up to room temperature and stirred for 12 h. The progress of the reaction was monitored by TLC. The solvents were removed *in vacuo* and the aqueous layer was extracted with CH_2Cl_2 (3×20 ml). The combined organic phases were dried over Na_2SO_4, filtered and concentrated to give the crude product. The crude product was further purified by flash column chromatography (CH_2Cl_2 then CH_2Cl_2/MeOH/Et_3N; 96:3:1) to afford **28** (0.11 g, 0.16 mmol, 34%) as a brown solid: mp 141-144°C. R_f = 0.52 (MeOH/CH_2Cl_2; 3:7). IR (neat): 2926, 1730, 1649, 1548, 1509, 1307, 1197, 1158, 750 cm^{-1}. ^{1}H-NMR (CD_3OD, 400 MHz): δ 8.24 (d, *J* = 8.24 Hz, 1H) ; 7.62 (d, *J* = 8.54 Hz, 1H) ; 7.43 (t, *J* = 7.37 Hz, 1H) ; 7.25 (t, *J* = 7.63 Hz, 1H) ; 6.07 (s, 2H) ; 4.62-4.51 (m, 3H) ; 4.02 (t, *J* = 5.82 Hz, 2H) ; 3.14 (d, *J* = 10.94 Hz, 2H) ; 2.84-2.80 (m, 2H) ; 2.55 (s, 3H) ; 2.44 (s, 6H) ; 2.40-2.33 (m, 5H) ; 2.28 (s, 6H) ; 2.10-1.96 (m, 3H) ; 1.75-1.70 (m, 2H) ; 1.60-1.13 (m, 4H) ; 1.14-1.11 (m, 2H). ^{13}C-NMR (CD_3OD, 100 MHz):δ 174.2 ; 164.2 ; 155.2 ; 146.9 ; 142.5 ; 142.3 ; 138.6 ; 132.6 ; 128.1 ; 124.0 ; 123.8 ; 123.2 ; 122.8 ; 111.0 ; 65.2 ; 56.1 ; 53.1 ; 41.3 ; 40.5 ; 33.1 ; 25.9 ; 25.6 ; 16.6 ; 14.6 ; 14.5. HRMS-ESI (+) *m/z* calcd for $C_{37}H_{48}BF_2N_6O_3$ 673.3849 $[M+H]^+$, found 673.3795 $[M+H]^+$. UV-Vis/Fluo (MeOH): $\lambda_{max\ abs}$ 497 nm, $\lambda_{max\ em}$ 505 nm. Purity HPLC 95%, t_R = 27.94 min.

5,5-Difluoro-7,9-dimethyl-1-(3-((3-(3-(((*endo*)-9-methyl-9azabicyclo[3.3.1]nonan-3-yl)carbamoyl)-1*H*-indazol-1-yl)propyl)amino)-3-oxopropyl)-5*H*-dipyrrolo[1,2-*c*:2',1'-*f*][1,3,2]diazaborinin-4-ium-5-uide (14)

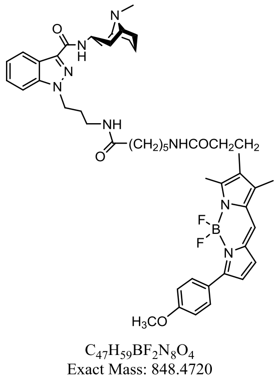

$C_{47}H_{59}BF_2N_8O_4$
Exact Mass: 848.4720

To a solution of amine **11**•2 HCl[13] (6.0 mg, 0.016 mmol) in dry DMF (0.5 ml) was added DIEA (4.1 mg, 0.032 mmol), the mixture was stirred for 10 min. Then a solution of BODIPY TMR-X succinimidyl ester (5.0 mg, 0.008 mmol) in DMF (1 ml) was added to the mixture and stirred at room temperature for 3.5 h. The progress of the reaction was monitored by TLC. DMF was removed *in vacuo* and the crude product was purified by flash column chromatography (CH_2Cl_2 then CH_2Cl_2/MeOH/Et_3N; 96:3:1) to afford **14** (6.7 mg, 0.008 mmol, 96%) as a pink solid: R_f = 0.09 (MeOH/CH_2Cl_2; 3:7). IR (neat): 3317, 2922, 2857, 1601, 1464, 1430, 1138, 1050, 1026, 749 cm^{-1}. ^{1}H NMR (CD_3OD, 400 MHz): δ 8.11 (d, *J* = 8.19 Hz, 1H) ; 7.75 (d, *J* = 8.87 Hz, 2H) ; 7.48 (d, *J* = 8.55 Hz, 1H) ; 7.33 (t, *J* = 7.24 Hz, 1H) ; 7.29 (s, 1H) ; 7.16 (t, *J* = 4.09 Hz, 1H) ; 6.93 (d, *J* = 4.09 Hz, 1H) ; 6.82 (d, *J* = 8.89 Hz, 2H) ; 6.46 (d, *J* = 4.08 Hz, 1H) ; 4.46 (tt, *J* = 6.78 Hz, *J* = 11.77 Hz, 1H) ; 4.53 (t, *J* = 6.77 Hz, 2H) ; 3.70 (s, 3H) ; 3.20-3.02 (m, 4H) ; 2.98 (t, *J* = 6.83 Hz, 2H) ; 2.63 (t, *J* = 7.13 Hz, 2H) ; 2.44 (s, 3H) ; 2.39-2.27 (m, 4H) ; 2.20 (t, *J* = 7.14 Hz, 2H) ; 2.12 (s, 3H) ; 1.99-

1.91 (m, 6H) ; 1.53-1.36 (m, 4H) ; 1.30-1.14 (m, 7H) ; 1.10-1.00 (m, 2H). ^{13}C NMR (CD_3OD, 100 MHz): δ 178.1 ; 174.2 ; 164.7 ; 144.0 ; 141.6 ; 139.0 ; 131.9 ; 128.4 ; 128.0 ; 124.7 ; 123.7 ; 123.2 ; 114.6 ; 110.9 ; 104.4 ; 55.8 ; 53.0 ; 47.8 ; 41.4 ; 40.5 ; 40.3 ; 37.9 ; 36.9 ; 36.8 ; 33.2 ; 30.3 ; 30.0 ; 29.2 ; 27.5 ; 26.6 ; 25.7 ; 14.8 ; 13.8 ; 10.3 ; 9.6. HRMS-ESI (+) *m/z* calcd for $C_{47}H_{60}BF_2N_8O_4$ 849.4799 $[M+H]^+$, found 849.4801 $[M+H]^+$. UV-Vis/Fluo (MeOH): $\lambda_{max\ abs}$ 536 nm, $\lambda_{max\ em}$ 573 nm. Purity HPLC 95%, t_R = 28.71 min.

1-(3-(7-(Diethylamino)-2-oxo-2*H*-chromene-3-carboxamido)propyl)-*N*-((3-endo)-9-methyl-9-azabicyclo[3.3.1]nonan-3-yl)-1*H*-indazole-3-carboxamide (15)

General procedure I: coupling to coumarin carboxylic acid.

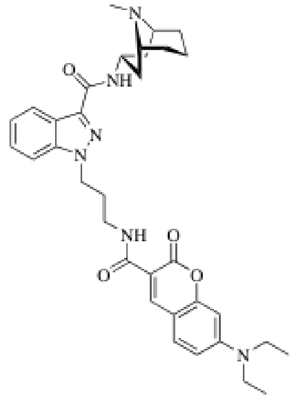

M. F. : $C_{34}H_{42}N_6O_4$; M. W. : 598.73

Coumarin carboxylic acid (0.03 g, 0.115 mmol, 1 eq.), DCC (0.03 g, 0.149 mmol, 1.3 eq.) and HOBt (0.015 g, 0.115 mmol, 1 eq.) were put together with a stirrer in a two-neck round bottom flask and dried under high vacuum for 1 h. This mixture was then dissolved in dry DMF/CH_2Cl_2 (1:4, 5 ml) and stirred for 2 h at room temperature under N_2 atmosphere. A solution of amine **11**•2 HCl (0.054 g, 0.126 mmol) mixed with Et_3N (0.032 ml, 0.23 mmol, 2 eq.) in dry CH_2Cl_2 (2 ml) was added dropwise to the mixture and left stirring for 24 h at RT. The solvents were removed *in vacuo* and the crude product was extracted with CH_2Cl_2 (3×15ml) and washed with $NaHCO_3$ sat. The combined organic layers were dried over Na_2SO_4, filtered and concentrated to yield the crude product. The crude product was further purified by flash chromatography on silica gel (CH_2Cl_2/MeOH/Et_3N, 96:3:1). The product was then washed with water (4 ml), $NaHCO_3$ sat. (3 ml) and extracted with $CHCl_3$ (3×15 ml) to remove triethylamine impurities and afforded **15** (0.046 g, 0.076 mmol, 66% yield) as a yellow solid: R_f = 0.18 (CH_2Cl_2/MeOH/Et_3N, 96:3:1). IR (film): 3360, 2925, 2605, 2500, 1695, 1645, 1615, 1580, 1535, 1510 cm^{-1}. ^1H-NMR (300 MHz, CD_3OD): δ 8,53 (s, 1H, arom. coum.) ; 8.19 (d, *J* = 8.2 Hz, 1H, arom. indaz.) ; 7.63 (d, *J* = 8.6 Hz, 1H, arom. coum.) ; 7.5 (d, *J* = 9.0 Hz, 1H, arom. indaz.) ; 7.48 – 7.41 (m, 1H, arom. indaz.) ; 7.3-7.2 (m, 1H, arom. indaz.) ; 6.81 (dd, *J* = 9.0, 2.4 Hz, 1H, arom. coum.) ; 6.46 (d, *J* = 2.4 Hz, 1H, arom. coum.) ; 4.62 (t, *J* = 6.2 Hz, 3H) ; 3.50 (q, *J* = 7.2 Hz, 4H, -N(C*H*$_2$CH$_3$)$_2$ coum.) ; 3.24 (m, *J* = 3.3, 1.6 Hz, 2H) ; 3.12 (d, *J* = 10.1 Hz, 2H) ; 2.73 (s, 3 H, -N*Me* granatane) ; 2.50 – 2.32 (m, 2H) ; 2.30 – 2.02 (m, 3H) ; 1.94 – 1.77 (m, 1H, granatane) ; 1.69 (t, *J* = 12.6 Hz, 2H) ; 1.45 – 1.32 (m, 1H) ; 1.31 – 1.08 (m, 8H, -N(CH$_2$C*H*$_3$)$_2$ coum., linker) ; 0.89 (t, *J* = 6.4 Hz, 1H). ^{13}C-NMR (75 MHz, CD_3OD): δ 165.2 ; 163.8 ; 159.0 ; 154.5 ; 149.3 ; 142.2 ; 132.5 ; 129.7 ; 127.9 ; 123.7 ; 123 ; 118.6 ; 117.5 ; 111.7 ; 110.9 ; 109.4 ; 97.1 ; 53.8 ; 45.8 ; 40.4 ; 39 ; 37.2 ; 32.4 ; 30.6 ; 29.5 ; 25.6 ; 14.1 ; 12.6 ; 9.5. MS (ESI): 599 $[M+H]^+$. HRMS-ESI: [M+H, $C_{34}H_{43}N_6O_4$]: calculated 599.3346, found 599.3345. UV-Vis/Fluo (MeOH): $\lambda_{max\ abs}$ 420 nm, $\lambda_{max\ em}$ 467 nm. Purity UPLC 98%, t_R = 2.16 min.

2-(6-Hydroxy-3-oxo-3*H*-xanthen-9-yl)-5-(3-(3-(3-(9-methyl-9-azabicyclo [3.3.1]nonan-3-yl)carbamoyl)-1*H*-indazol-1-yl)propyl)thioureido)benzoic acid (16)

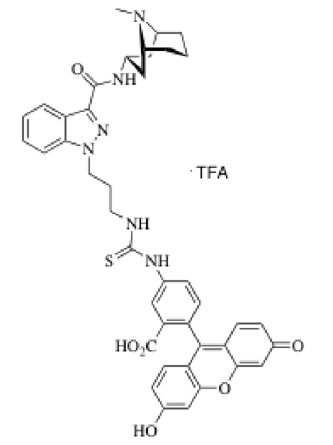

M. F. : $C_{43}H_{41}F_3N_6O_8S$; M. W. : 858.88

To a stirred solution of amine **11**•2 HCl (0.032 g, 0.074 mmol, 1 eq.) in DMF (1 ml) was added Et_3N (61 µl, 0.294 mmol, 6 eq.) and fluorescein isothiocyanate (0.03 g, 0.077 mmol, 1.05 eq.). The mixture was reacted for 4.5 h at room temperature. The solvent was removed under high vacuum. The crude product was purified by prep-LC, using a gradient of 100% A (100% water with 0.1% of trifluoroacetic acid) to 100% D (40% water with 60% acetonitrile and 0.1% trifluoroacetic acid). The product had a retention time of 22 min and was eluted at 45% A-55% D. The fractions collected were dried by lyophilization to give **16•**TFA as an orange solid (0.023 g, 0.0267 mmol, 36% yield): IR (neat): 2620, 1670, 1590, 1535, 1455, 1275, 1175, 1115, 840 cm^{-1}. ^{1}H-NMR (300 MHz, CD_3OD): δ 8.22 (d, *J* = 8.2 Hz, 1H, indaz.) ; 8.19 (s, 1H, fluoresc.) ; 7.71 (d, *J* = 8.5 Hz, 1H, indaz.) ; 7.66 (d, *J* = 1.9 Hz, 1H, fluoresc.) ; 7.49 (pseudo-t, *J* = 7.3 Hz, 1H, indaz.) ; 7.31 (pseudo-t, *J* = 7.5 Hz, 1H, indaz.) ; 7.17 (d, *J* = 8.3 Hz, 1H, fluoresc.) ; 6.79 – 6.63 (m, 4H, fluoresc.) ; 6.58 (dd, *J* = 8.7, 2.3 Hz, 2H, fluoresc.) ; 4.70 – 4.56 (m, 3H) ; 3.72 (d, *J* = 10.4 Hz, 3H) ; 2.96 (s, 3H) ; 2.71 – 2.48 (m, 2H) ; 2.43 – 2.28 (m, 2H) ; 2.27 – 2.10 (m, 2H) ; 2.09 – 1.90 (m, 3H) ; 1.73 – 1.47 (m, 3H) ; 1.32 (d, *J* = 12.1 Hz, 1H). ^{13}C-NMR (75 MHz, CD_3OD): δ 183 ; 171.1 ; 164.5 ; 161.9 ; 154.5 ; 142.3 ; 137.8 ; 130.3 ; 129 ; 128 ; 125.8 ; 123.9 ; 122.9 ; 113.8 ; 111.7 ; 110.9 ; 103.5 ; 55.1 ; 47.6 ; 46.9 ; 42.9 ; 39.1 ; 38.1 ; 32.3 ; 29.6 ; 24.1 ; 12.5. MS (ESI): 745 $[M–TFA+H]^+$. HRMS-ESI: [M–TFA+H, $C_{41}H_{41}N_6O_6S$]: calculated 745.2803, found 745.2817. UV-Vis/Fluo (MeOH): $\lambda_{max\ abs}$ 481 nm, $\lambda_{max\ em}$ 520 nm. Purity UPLC 95%, t_R = 1.35 min.

Synthesis of linker building block 9:

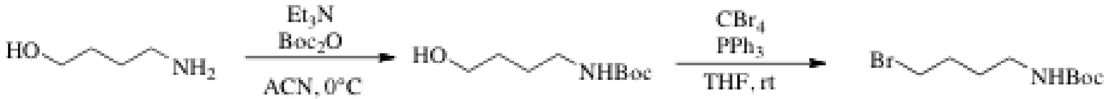

***tert*-Butyl (4-hydroxybutyl)carbamate**

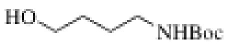

M. F. : $C_9H_{19}NO_3$; M. W. : 183.13

4-amino-1-butanol (0.5 g, 5.61 mmol) was dissolved in acetonitrile (56 ml). The solution was cooled to 0°C and Et_3N (1.56 ml, 11.22 mmol, 2 eq.) was added followed by Boc_2O (1.346 g, 6.17 mmol). The reaction was followed by TLC and quenched with water (20 ml) after 6.5 h. The solution was extracted with EtOAc (3×30ml), brine was used to help phase separation. The combined organic layers were dried over Na_2SO_4, filtered and concentrated under reduced pressure. The crude product was purified by flash chromatography on silica gel (CH_2Cl_2/MeOH, 95:5) to give the title compound as a colorless oil (1.06 g, quantitative yield): R_f = 0.3 (CH_2Cl_2/MeOH, 95:5). IR (film): 3345, 2935, 1680, 1525, 1455, 1390, 1365, 1250, 1165, 1055, 1040 cm^{-1}. ^{1}H-NMR (300 MHz, $CDCl_3$): δ 4.61 (s broad, 1H, -N*H*Boc) ;

3.68 (q broad, J = 6.0 Hz, 2H, -CH_2OH) ; 3.15 (q broad, J = 5.5 Hz, 2H, -CH_2NHBoc) ; 1.59 (m, 4H, -(CH_2)$_2$-) ; 1.44 (s, 9H, Boc). ^{13}C-NMR (75 MHz, $CDCl_3$): δ 156.4 (amide, Boc) ; 79.3 (Boc) ; 62 (CH$_2$OH) ; 40.1 (CH$_2$NHBoc) ; 29.4; 28.3 (Boc) ; 26.2. MS (ESI): 212 $[M+Na]^+$. HRMS-ESI: [M+Na, $C_9H_{19}O_3NNa$]: calculated 212.1263, found 212.1260.

tert-Butyl (4-bromobutyl)carbamate (9)[S1]

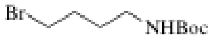

M. F. : $C_9H_{18}BrNO_2$; M. W. : 252.14

tert-Butyl (4-hydroxybutyl)carbamate (1.06 g, 5.788 mmol) was dissolved in dry THF (54 ml) followed by addition of Ph_3P (2.86 g, 10.92 mmol, 1.9 eq.). Then CBr_4 (3.62 g, 10.92 mmol, 1.9 eq.) was slowly added to the mixture. After 3 h the solution was filtered through a celite pad to eliminate the by-products and washed with Et_2O. The solvents were removed under high vacuum. The crude product was purified by flash chromatography on silica gel (hexane/EtOAc, 3:1) to give **9** as a white solid at 5°C (1.73 g, quantitative yield): R_f = 0.7 (hexane/EtOAc, 3:1). IR (film): 3350, 2975, 2945, 1695, 1510, 1455, 1390, 1365, 1250, 1165, 1005 cm^{-1}. ^{1}H-NMR (300 MHz, $CDCl_3$): δ 4.56 (s broad, 1H, NHBoc) ; 3.41 (t, J = 6.7 Hz, 2H, -CH_2Br) ; 3.13 (q broad, J = 5.9 Hz, 2H, -CH_2NHBoc) ; 1.94 – 1.79 (m, 2H, -CH_2-) ; 1.69 – 1.54 (m, 2H, -CH_2-) ; 1.43 (s, 9H, Boc). ^{13}C-NMR (75 MHz, CD_3OD): δ 156.0 (amide, Boc) ; 79.4 (Boc) ; 66.6 (CH$_2$OH) ; 39.8 (CH$_2$NHBoc) ; 33.4 (CH$_2$Br) ; 30.0; 28.9; 28.4 (Boc). MS (ESI): 274 $[M+Na]^+$. HRMS-ESI: [M+Na, $C_9H_{18}BrNO_2Na$]: calculated 274.0413, found 274.0410.

tert-Butyl (4-(3-(((3-*endo*)-9-methyl-9-azabicyclo[3.3.1]nonan-3-yl)carbamoyl)-1*H*-indazol-1-yl)butyl)carbamate

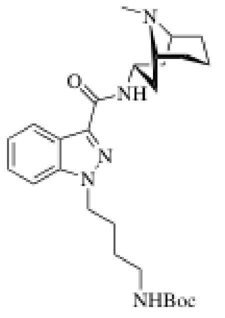

M. F. : $C_{26}H_{39}N_5O_3$; M. W. : 469.61

Amide **7** (0.215 g, 0.721 mmol) was dissolved in DMF/THF (1:5, 7.2 ml). The reaction was cooled to 0°C and stirred for 5 min. A solution of *t*-BuOK (0.089 g, 0.793 mmol, 1.1 eq) in dry THF (2ml) was added at 0°C and stirred 15 min. Then linker **9** (0.218 g, 0.865 mmol) in dry THF (2 ml) was added. The mixture was brought to room temperature and stirred overnight. Progress of the reaction was monitored by TLC. The solvents were removed *in vacuo*, water (20 ml) was added and the solution was extracted with EtOAc (3×20 ml). The combined organic layers were dried over Na_2SO_4, filtered and concentrated under reduced pressure. The crude product was purified by crystallization (MeOH/Et_2O) to give the title compound as a white solid (0.265 g, 78% yield): R_f = 0.43 (CH_2Cl_2/MeOH, 2:1). IR (film): 2930, 2360, 1695, 1655, 1535, 1365, 1170, 750 cm^{-1}. ^{1}H-NMR (300 MHz, CD_3OD): δ 8.22 (d, J = 8.2 Hz, 1H, indaz.) ; 7.60 (d, J = 7.6 Hz, 1H, indaz.) ; 7.41 (t, J = 6.9 Hz, 1H, indaz.) ; 7.24 (t, J = 7.2 Hz, 1H, indaz.) ; 4.66 – 4.39 (m, 3H) ; 3.43 (t, J = 6.0 Hz, 1H) ; 3.29 (m, 1H) ; 3.17 – 2.99 (m, 4H) ; 2.51 (s, 3H, -NCH_3) ; 2.48 – 2.34 (m, 2H) ; 2.21 – 1.88 (m, 5H) ; 1.90 – 1.74 (m, 2H) ; 1.58 (m, 3H) ; 1.40 (s, 9 H, Boc). ^{13}C-NMR (75 MHz, CD_3OD): δ 164.0; 158.3 (amide, Boc) ; 142.1; 138.1; 127.6 ; 123.4 ; 123.1 ; 110.7 ;

[S1] Maruyoshi, K.; Nonaka, K.; Sagane, T.; Demura, T.; Yamaguchi, T.; Matsumori, N.; Oishi, T; Murata, M. *Chem. Eur. J.* **2009**, *15*, 1618.

79.8 (Boc) ; 52.7 ; 49.9; 41.1 ; 40.2 ; 34.5 ; 33.1; 31.0 ; 28.7 ; 28.1 ; 27.9; 25.4; 14.8. MS (ESI): 470 [M+H]$^+$. HRMS-ESI: [M+H, $C_{26}H_{40}O_3N_5$]: calculated 470.3126, found 470.3129.

1-(4-Aminobutyl)-*N*-(9-methyl-9-azabicyclo[3.3.1]nonan-3-yl)-1*H*-indazole-3-carboxamide dihydrochloride (12)

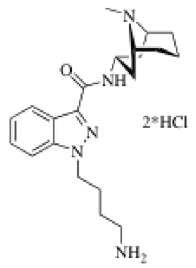

M. F. : $C_{21}H_{33}Cl_2N_5O$; M. W. : 442.42

To a solution of *tert*-butyl (4-(3-(((3-*endo*-)-9-methyl-9-azabicyclo[3.3.1]nonan-3-yl)carbamoyl)-1*H*-indazol-1-yl)butyl)carbamate (0.192 g, 0.409 mmol) in MeOH (8 ml) was added drop wise a solution of 1.2M HCl in MeOH (10 ml) the reaction was stirred overnight at room temperature. The solvents were removed *in vacuo* and three times co-evaporated with toluene. The crude product was further purified by crystallization (MeOH/Et_2O) to afford amine **12**•2 HCl (0.079 g, 0.18 mmol, 44% yield) as a white solid: IR (film): 3395, 2940, 2360, 2340, 1635, 1540, 1480, 1445, 1310, 1190, 1135, 1005 cm^{-1}. ^{1}H-NMR (300 MHz, CD_3OD): δ 8.22 (d, *J* = 8.1 Hz, 1H, indaz.) ; 7.69 (d, *J* = 8.5 Hz, 1H, indaz.) ; 7.49 (t, *J* = 7.5 Hz, 1H, indaz.) ; 7.31 (t, *J* = 7.7 Hz, 1H, indaz.) ; 4.75 – 4.39 (m, 3H) ; 3.75 (d, *J* = 10.0 Hz, 2H) ; 3.02 – 2.85 (m, 4H) ; 2.77 – 2.48 (m, 2H) ; 2.40 – 2.15 (m, 3H) ; 2.14 – 1.89 (m, 5H) ; 1.77 – 1.49 (m, 5H). ^{13}C-NMR (75 MHz, CD_3OD): δ 164.3 (arom.) ; 142.2 (arom.) ; 138 (arom.) ; 128 (arom.) ; 123.8 (arom.) ; 122.8 (arom.) ; 111.1 (arom.) ; 56 ; 55.2 ; 49.4; 40.4 ; 39.2 ; 38.6 ; 32 ; 27.7 ; 25.8; 24.2; 12.7. MS (ESI): 370 [M–2HCl+H]$^+$. HRMS-ESI: [M–2HCl+H, $C_{21}H_{32}ON_5$]: calculated 370.2601, found 370.2612.

1-(4-(7-(Diethylamino)-2-oxo-2*H*-chromene-3-carboxamido)butyl)-*N*-(9-methyl-9-azabicyclo[3.3.1]nonan-3-yl)-1*H*-indazole-3-carboxamide (17)

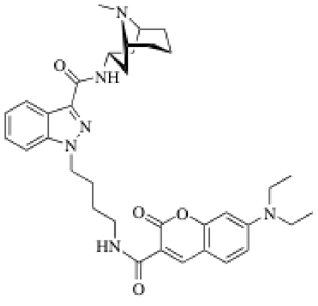

M. F. : $C_{35}H_{44}N_6O_4$; M. W. : 612.76

Following procedure I; coumarin carboxylic acid (0.02 g, 0.079 mmol), DCC (0.021 g, 0.103 mmol, 1.3 eq.) and HOBt (0.01 g, 0.079 mmol, 1 eq.) were mixed in a two-neck round bottom flask in DMF/CH_2Cl_2. A solution of amine **12**•2 HCl (0.032 g, 0.087 mmol) mixed with Et_3N (0.02ml, 0.16 mmol, 2 eq.) in dry CH_2Cl_2 (1.3 ml) was then added drop-wise. After work-up, the combined organic layers were dried over Na_2SO_4, filtered and concentrated to yield the crude product. The crude product was further purified by flash chromatography on silica gel (CH_2Cl_2/MeOH/Et_3N, 96:3:1). The product was then washed with water (4 ml), $NaHCO_3$ sat. (3 ml) and extracted with $CHCl_3$ (3×15 ml) to remove triethylamine impurities and to give fluorescent ligand **17** (0.039 g, 0.064 mmol, 80% yield) as a yellow solid: R_f = 0.63 (CH_2Cl_2/MeOH, 7:3). IR (film): 3360, 2360, 2340, 1510, 1695, 1645, 1615, 1580, 1535, 1510 cm^{-1}. ^{1}H-NMR (300 MHz, CD_3OD): δ 8.53 (s, 1H, coumarin) ; 8.19 (d, *J* = 8.2 Hz, 1H, indazole) ; 7.62 (d, *J* = 8.5 Hz, 1H, coum.) ; 7.48 (d, *J* = 9.0 Hz, 1H, indaz.) ; 7.42 (dd, *J* = 11.3, 4.1 Hz, 1H, indaz.) ; 7.22 (dd, *J* = 11.3, 4.1 Hz, 1H, indaz.) ; 6.76 (dd, *J* = 9.0, 2.0 Hz, 1H, coum.) ; 6.48 (s, 1H, coum.) ; 4.55 (tt, *J* = 11.6, 6.8 Hz, 1H) ; 4.51 (t, *J* = 6.7 Hz, 2H) ; 3.48 (q, *J* = 7.0 Hz, 4H, -N(C*H*$_2$CH$_3$)$_2$ coum.) ; 3.40 (t, *J* = 6.8 Hz, 2H) ; 3.08 (d, *J* = 10.6 Hz, 2H) ; 2.50 (s,

3H, -NCH_3) ; 2.47 – 2.34 (m, 2H) ; 2.21 – 1.90 (m, 5H) ; 1.65 – 1.52 (m, 4H) ; 1.34 – 1.26 (m, 1H) ; 1.21 (t, J = 7.1 Hz, 6H, -N(CH_2CH_3)$_2$ coum.) ; 1.15 – 1.03 (m, 2H). ^{13}C-NMR (75 MHz, $CDCl_3$): δ 163.4 ; 162.9 ; 162.2 ; 157.8 ; 152.7 ; 148.2 ; 141.0 ; 137.8 ; 131.3 ; 126.8 ; 123.2 ; 123.0 ; 122.5 ; 110.3 ; 110.1 ; 109.2 ; 108.5 ; 96.7 ; 51.7 ; 49.2 ; 48.9 ; 45.2 ; 40.8 ; 40.7 ; 38.9 ; 34.1 ; 32.8 ; 29.8 ; 27.2 ; 27.0 ; 25.7 ; 25.2 ; 25.1 ; 14.4 ; 12.5. MS (ESI): 613 $[M+H]^+$. HRMS-ESI: [M+H, $C_{35}H_{45}N_6O_4$]: calculated 613.3497, found 613.3515. UV-Vis/Fluo (MeOH): $\lambda_{max\ abs}$ 420 nm, $\lambda_{max\ em}$ 465 nm. Purity UPLC 96%, t_R = 2.20 min

3-(1-(4-(3-(3-carboxy-4-(6-hydroxy-3-oxo-3*H*-xanthen-9-yl)phenyl) thioureido)butyl)-1*H*-indazole-3-carboxamido)-9-methyl-9-azabicyclo[3.3.1] nonan-9-ium 2,2,2-trifluoroacetate (18)

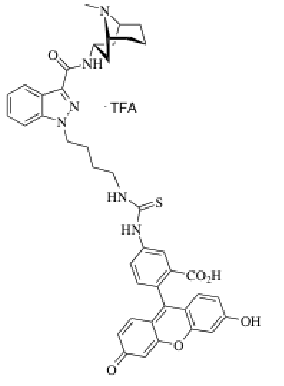

M. F. : $C_{44}H_{43}F_3N_6O_8S$; M. W. : 872.9

To a stirred solution of amine **12•**2 HCl (0.03 g, 0.068 mmol, 1 eq.) in DMF (0.5 ml) was added Et_3N (47 µl, 0.339 mmol, 5 eq.) and fluorescein isothiocyanate (0.029 g, 0.075 mmol, 1.1 eq.). The mixture was reacted for 5 h at RT. The solvent was removed under high vacuum. The crude product was purified by prep-LC using a gradient of 60% A (100% water with 0.1% of trifluoroacetic acid) to 100% D (40% water with 60% acetonitrile and 0.1% trifluoroacetic acid) in 55 min. The product had a retention time of 11 min and was eluted at 53% A-47% D. The fractions collected were dried by lyophilization to give **18•**TFA as an orange solid (0.025 g, 0.0286 mmol, 42% yield): IR (neat): 1590, 1540, 1455, 1270, 1175, 1115, 835, 800 cm^{-1}. ^{1}H-NMR (300 MHz, CD_3OD): δ 8.21 (d, J = 8.2 Hz, 1H, indaz.) ; 8.13 (s, 1H, fluoresc.) ; 7.70 (d, J = 8.4 Hz, 1H, indaz.) ; 7.67 (s, 1H, fluoresc.) ; 7.48 (pseudo-t, J = 7.2 Hz, 1H, indaz.) ; 7.30 (pseudo-t, J = 7.6 Hz, 1H, indaz.) ; 7.15 (d, J = 8.2 Hz, 1H, fluoresc.) ; 6.76 – 6.68 (m, 4H, fluoresc.) ; 6.58 (dd, J = 8.7, 2.0 Hz, 2H, fluoresc.) ; 4.70 – 4.56 (m, 3H) ; 3.72 (d, J = 10.4 Hz, 3H) ; 2.96 (s, 3H) ; 2.71 – 2.48 (m, 2H) ; 2.43 – 2.28 (m, 2H) ; 2.27 – 2.10 (m, 2H) ; 2.09 – 1.90 (m, 3H) ; 1.73 – 1.47 (m, 3H) ; 1.32 (d, J = 12.1 Hz, 1H). HSQC ^{13}C-NMR (100 MHz, CD_3OD): δ 183 ; 171.1 ; 164.5 ; 161.9 ; 154.5 ; 142.3 ; 137.8 ; 131.3 ; 129.8 ; 127.6 ; 125.4 ; 123.3 ; 122.4 ; 119.6 ; 113.2 ; 110.5 ; 103.0 ; 54.6 ; 49.4 ; 44.2 ; 41.5 ; 38.0 ; 37.6 ; 31.6 ; 30.4 ; 27.6 ; 27 ; 23.8 ; 12.1. MS (ESI): 759 $[M\text{–}TFA+H]^+$. HRMS-ESI: [M–TFA+H, $C_{42}H_{43}N_6O_6S$]: calculated 759.2959, found 759.2968. UV-Vis/Fluo (Phosphate buffer pH 7): $\lambda_{max\ abs}$ 493 nm, $\lambda_{max\ em}$ 519 nm. Purity UPLC 93%, t_R = 1.88 min.

***N*-((*endo*)-9-methyl-9-azabicyclo[3.3.1]nonan-3-yl)-1-(4-((7-nitrobenzo[*c*][1,2,5] oxadiazol-4-yl)amino)butyl)-1*H*-indazole-3-carboxamide (19)**

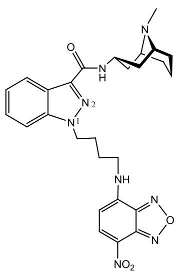

Chemical Formula: $C_{27}H_{32}N_8O_4$
Exact Mass: 532.25

To a solution of **12•**2 HCl (0.1 g, 0.23 mmol) in DMF/THF/H_2O (1:4:2, 6.0 ml) solid Na_2CO_3 was added and stirred at room temperature for 10 min. Then a solution of NBD chloride (0.09 g, 0.45 mmol) in THF (2 ml) was added to the mixture and stirred at 45 °C for 12 h. The solvents were removed *in vacuo* and the crude product was purified by flash column chromatography (CH_2Cl_2 then CH_2Cl_2/MeOH/Et_3N; 96:3:1) to afford **19** (0.072 g, 0.13 mmol, 60%) as a brown solid: mp 142-145°C. R_f = 0.23 (MeOH/CH_2Cl_2; 3:7). IR (neat): 2923, 1620, 1579, 1529, 1290, 1254, 1173, 750, 738 cm^{-1}. ^{1}H-

NMR (CD_3OD, 400 MHz): δ 8.36 (d, *J* = 8.87 Hz, 1H) ; 8.16 (d, *J* = 8.20 Hz, 1H) ; 7.62 (d, *J* = 8.54 Hz, 1H) ; 7.40 (t, *J* = 7.66 Hz, 1H) ; 7.22 (t, *J* = 7.62 Hz, 1H) ; 6.14 (d, *J* = 8.87 Hz, 1H) ; 4.59-4.56 (m, 3H) ; 3.50 (br, 2H) ; 3.12 (d, 2H, *J* = 10.81 Hz) ; 2.54 (s, 3H) ; 2.49-2.41 (m, 2H) ; 2.20-2.02 (m, 5H) ; 1.59-1.50 (m, 3H) ; 1.15-1.11 (m, 2H). ^{13}C-NMR (CD_3OD, 100 MHz): δ 164.2 ; 142.4 ; 138.5 ; 138.3 ; 128.0 ; 123.9 ; 123.9 ; 123.6 ; 123.2 ; 110.9 ; 99.7 ; 60.1 ; 52.9 ; 49.8 ; 47.2 ; 41.5 ; 40.6 ; 33.3 ; 27.9 ; 25.7 ; 14.9. HRMS-ESI (+) *m/z* calcd for $C_{27}H_{33}N_8O_4$ 533.2626 $[M+H]^+$, found 533.2619 $[M+H]^+$. UV-Vis/Fluo (MeOH): $\lambda_{max\ abs}$ 465 nm, $\lambda_{max\ em}$ 536 nm. Purity HPLC 95%, t_R = 20.19 min.

Synthesis of linker building block 10: [S2]

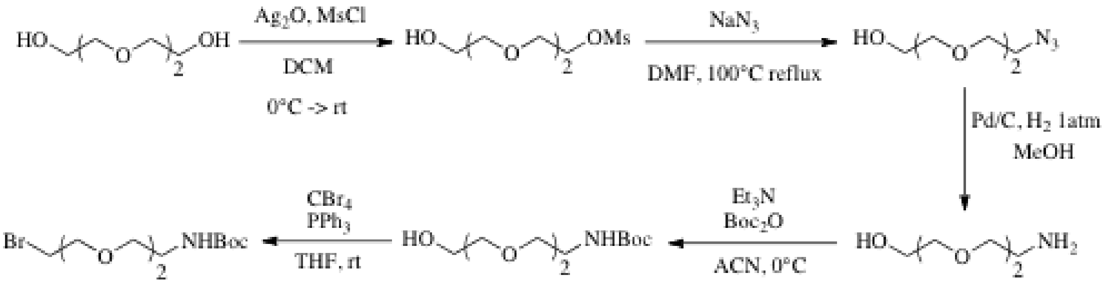

2-(2-(2-hydroxyethoxy)ethoxy)ethyl methanesulfonate

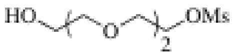

M. F. : $C_7H_{16}O_6S$; M. W. : 228.26

Triethylene glycol (4 g, 26.64 mmol, 3.6 ml) was dissolved in dry CH_2Cl_2 (62 ml). The solution was cooled to 0°C before adding silver(I)oxide (6.8 g, 29.3 mmol, 1.1 eq.), followed by MsCl (2.5 ml, 31.97 mmol, 1.2 eq.). The mixture was stirred at room temperature for 5 d. Progress of the reaction was followed by TLC. It was then filtered through celite and the solvent was evaporated. The crude product was purified by flash chromatography on silica gel (EtOAc/MeOH, 95:5). The bis-mesylated product eluted first followed by the title compound. Colorless oil (4.54 g, 75% yield): R_f = 0.58 (EtOAc/MeOH, 95:5). IR (film): 3450, 2875, 2165, 1350, 1175 cm^{-1}. ^1H-NMR (300 MHz, $CDCl_3$): δ 4.40 – 4.29 (m, 2H) ; 3.78 – 3.72 (m, 2H) ; 3.72 – 3.67 (m, 2H) ; 3.65 (s, 4H) ; 3.60 – 3.52 (m, 2H) ; 3.05 (s, 3H, -C*H_3*). ^{13}C-NMR (75 MHz, $CDCl_3$): δ 72.5; 70.6; 70.3; 69.0; 61.7; 37.7. MS (ESI): 229 $[M+H]^+$. HRMS-ESI: [M+H, $C_7H_{17}O_6S$]: calculated 229.0740, found 229.0741.

2-(2-(2-azidoethoxy)ethoxy)ethanol

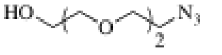

M. F. : $C_6H_{13}N_3O_3$; M. W. : 175.18

2-(2-(2-hydroxyethoxy)ethoxy)ethylmethane sulfonate (4.54 g, 19.8 mmol) was dissolved in DMF (50 ml) under stirring. NaN_3 (1.93 g, 29.7 mmol, 1.5 eq.) was added and the mixture was heated at 100°C under reflux for 18 h. Progress of the reaction was followed by TLC. The solution was cooled to room temperature and water (25 ml) was added, the aqueous phase was extracted with EtOAc (3×25 ml). The combined organic layers were washed with water (25 ml) and brine (25 ml). They were dried over $MgSO_4$, filtered and concentrated under reduced pressure to give the

[S2] Svedhem, S.; Hollander, C.-Å.; Shi, J.; Konradsson, P.; Liedberg, B; Svensson, S. C. T. *J. Org. Chem.* **2001**, *66*, 4494.

crude product. The crude product was further purified by flash chromatography on silica gel (EtOAc/MeOH, 98:2) to give the title compound as an oil (2g, 58% yield): R_f = 0.75 (CH_2Cl_2/MeOH, 9.6:0.4). IR (film): 3395, 2920, 2870, 2100, 1670, 1445, 1385, 1350, 1290, 1120, 1065, 930 cm^{-1}. ^{1}H-NMR (300 MHz, $CDCl_3$): δ 3.74 – 3.70 (m, 2H) ; 3.69 – 3.64 (m, 6H) ; 3.62 – 3.57 (m, 2H) ; 3.41 – 3.35 (t, 2H) ; 2.39 (s broad, 1H, -O*H*). ^{13}C-NMR (75 MHz, $CDCl_3$): δ 72.5; 70.7; 70.5; 70.1; 61.8; 50.7. MS (ESI): 198 $[M+Na]^+$. HRMS-ESI: [M+Na, $C_6H_{13}N_3O_3Na$]: calculated 198.0849, found 198.0851.

2-(2-(2-aminoethoxy)ethoxy)ethanol

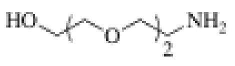

M. F. : $C_6H_{15}NO_3$; M. W. : 149.18

2-(2-(2-azidoethoxy)ethoxy)ethanol (0.7 g, 3.99 mmol) was dissolved in MeOH (9 ml), then palladium on activated carbon (0.196 g) was added under stirring. The flask was submitted to 3 evacuation/H_2 flushing cycles in order to establish the hydrogen atmosphere in the flask. The reaction was followed by TLC and showed completion after 8 h. The solution was filtered through celite to remove the Pd/C. The solution was concentrated under reduced pressure to give the title compound as an oil (0.6 g, quantitative yield): IR (film): 3365, 2860, 2115, 1665, 1455, 1350, 1115, 1065 cm^{-1}. ^{1}H-NMR (300 MHz, $CDCl_3$): δ 3.68 – 3.62 (m, 2H) ; 3.62 – 3.57 (m, 4H) ; 3.57 – 3.50 (m, 2H) ; 3.47 (t, J = 5.2 Hz, 2H) ; 2.78 (t, J = 5.1 Hz, 2H, -NH_2) ; 2.69 (s broad, 1H, -O*H*). ^{13}C-NMR (75 MHz, $CDCl_3$): δ 73.4; 73.2; 70.8; 70.6; 61.9; 41.8. MS (ESI): 150 $[M+H]^+$. HRMS-ESI: [M+H, $C_6H_{16}NO_3$]: calculated 150.1125, found 150.1120.

***tert*-Butyl (2-(2-(2-hydroxyethoxy)ethoxy)ethyl)carbamate**

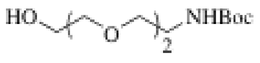

M. F. : $C_{11}H_{23}NO_5$; M. W. : 249.3

2-(2-(2-aminoethoxy)ethoxy)ethanol (0.6 g, 4.062 mmol) was dissolved in acetonitrile (25 ml). The solution was cooled to 0°C and Et_3N (1.13 ml, 8.12 mmol, 2 eq.) was added followed by Boc_2O (0.268 g, 1.23 mmol). The reaction was followed by TLC and stirred overnight at room temperature. Water (15 ml) was added and the solution was extracted with EtOAc (3×30 ml), brine was used to help separating the two layers (5 ml) and also (30 ml) for washing. The combined organic layers were dried over Na_2SO_4, filtered and concentrated under reduced pressure. The crude product was purified by flash chromatography on silica gel (CH_2Cl_2/MeOH, 96:4) to give the title compound as a yellow oil (0.670 g, 66 % yield): R_f = 0.46 (CH_2Cl_2/MeOH, 9.6:0.4). IR (film): 3335, 2920, 1710, 1530, 1455, 1365, 1255, 1170 cm^{-1}. ^{1}H-NMR (300 MHz, $CDCl_3$): δ 5.17 (s broad, 1 H, -N*H*Boc.) ; 3.75 – 3.69 (m, 2H) ; 3.66 – 3.56 (m, 6H) ; 3.53 (t, J = 5.2 Hz, 2H) ; 3.28 (d, J = 3.2 Hz, 2H) ; 2.8 (s broad, 1H, -O*H*) ; 1.42 (s, 9H, Boc). ^{13}C-NMR (75 MHz, $CDCl_3$): δ 156.1 (Boc) ; 79.3 (Boc) ; 72.7; 70.3; 61.7; 40.3; 28.3 ((CH_3)$_3$ Boc). MS (ESI): 272 $[M+Na]^+$. HRMS-ESI: [M+Na, $C_{11}H_{23}NO_5Na$]: calculated 272.1468, found 272.1473.

***tert*-Butyl (2-(2-(2-bromoethoxy)ethoxy)ethyl)carbamate (10)**

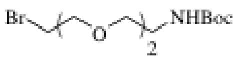

M. F. : $C_{11}H_{22}BrNO_4$; M. W. : 312.2

tert-butyl(2-(2-(2-hydroxyethoxy)ethoxy)ethyl)carbamate (0.5 g, 2.03 mmol) was dissolved in dry THF (20 ml) then Ph_3P (1.06 g, 4.06 mmol, 2 eq.) was added. CBr_4 (1.35 g, 4.06 mmol, 2 eq.) was slowly added to the mixture. After 3 h the

solution was filtered through celite to eliminate the phosphine oxide and washed with Et_2O. The solvents were removed under high vacuum. The crude product was purified by flash chromatography on silica gel (hexane/AcOEt, 4:1) to give **10** as a colorless oil (0.601 g, 95% yield): R_f = 0.42 (hexane/AcOEt, 4:1). IR (film): 3365, 2970, 2925, 2090, 1770, 1710, 1505, 1455, 1390, 1365, 1275, 1250, 1170, 1115, 1040 cm^{-1}. ^1H-NMR (300 MHz, $CDCl_3$): δ 5.00 (broad s, 1H, -N*H*Boc) ; 3.79 (t, *J* = 6.3 Hz, 2H) ; 3.67 – 3.58 (m, 4H) ; 3.53 (t, *J* = 5.2 Hz, 2H) ; 3.46 (t, *J* = 6.3 Hz, 2H) ; 3.30 (dd, *J* = 10.5, 5.2 Hz, 2H) ; 1.42 (s, 9H). ^{13}C-NMR (75 MHz, $CDCl_3$): δ 156.0 (Boc) ; 79.3 (Boc) ; 71.2; 70.5; 70.3; 70.2; 40.4; 30.3; 28.5 ((CH_3)$_3$ Boc). MS (ESI): 334 $[M+Na]^+$. HRMS-ESI: [M+Na, $C_{11}H_{22}BrNO_4Na$]: calculated 334.0624, found 334.0631.

***tert*-Butyl (2-(2-(2-(3-(9-methyl-9-azabicyclo[3.3.1]nonan-3-yl) carbamoyl) -1*H*-indazol-1-yl)ethoxy)ethoxy)ethyl)carbamate**

General procedure II: binding of linker to the granisetron core. [S3]

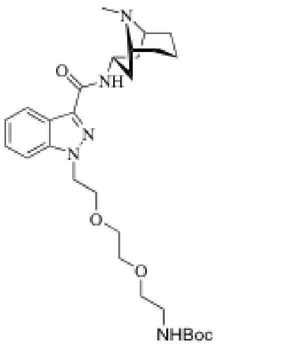

M. F. : $C_{28}H_{43}N_5O_5$; M. W. : 529.67

To a stirred solution of amide **7** (0.057 g, 0.19 mmol, 1 eq.) in DMF (2 ml), linker **10** (0.095 g, 0.3 mmol, 1.6 eq.) was added, followed by K_2CO_3 (0.047 g, 0.34 mmol, 1.8 eq.) and *n*-Bu_4NI (0.007 g, 0.019 mmol, 10 mol%) at RT. After stirring overnight at 60°C, the reaction mixture was partitioned between EtOAc-water, the organic layer was washed with sat. NH_4Cl, sat. $NaHCO_3$ and brine, dried over $MgSO_4$ and filtered. The solvent was removed *in vacuo*. The title compound was obtained as a white solid (0.102 g, 0.193 mmol, quantitative yield) and was used in the next step without further purification: IR (film): 3340, 2930, 2865, 1705, 1650, 1530, 1490, 1365, 1285, 1250, 1170, 1120 cm^{-1}. ^1H-NMR (300 MHz, CD_3OD): δ 8.22 (d, *J* = 8.2 Hz, 1H, indaz.) ; 7.66 (d, *J* = 8.6 Hz, 1H, indaz.) ; 7.45 (dd, *J* = 15.1, 0.7 Hz, 1H, indaz.) ; 7.27 (dd, *J* = 15.1, 0.7 Hz, 1H, indaz.) ; 4.66 (t, *J* = 5.2 Hz, 2H) ; 4.57 (tt, *J* = 11.6, 5.9 Hz, 1H) ; 3.98 (t, *J* = 5.2 Hz, 2H) ; 2.53 (s, 3H) ; 2.46 (m, 2H) ; 2.24 – 1.93 (m, 2H) ; 1.70 – 1.51 (m, 3H) ; 1.43 (s, 9H) ; 1.16 (d, *J* = 12.7 Hz, 2H). ^{13}C-NMR (75 MHz, CD_3OD): δ 162.5 ; 156.4 ; 147.4 ; 142.1 ; 138.6 ; 132.7 ; 132.6 ; 132.5 ; 129.1 ; 128.9 ; 127.3 ; 123.4 ; 123.3 ; 123 ; 110.2 ; 79.7 ; 71.7 ; 71.3 ; 71.0 ; 70.7 ; 70.4 ; 51.9 ; 50.0 ; 41.3 ; 41.1 ; 33.4 ; 30.7 ; 28.9 ; 25.4 ; 14.8. MS (ESI): 530 $[M+H]^+$. HRMS-ESI: [M+H, $C_{28}H_{44}N_5O_5$]: calculated 530.3337, found 530.3339.

[S3] Kan, T.; Kita, Y.; Morohashi, Y.; Tominari, Y.; Hosoda, S.; Tomita, T.; Natsugari, H.; Iwatsubo, T.; Fukuyama T. *Org. Lett.***2007**, *9*, 2055.

1-(2-(2-(2-Aminoethoxy)ethoxy)ethyl)-*N*-(9-methyl-9-azabicyclo[3.3.1] nonan-3-yl)-1*H*-indazole-3-carboxamide dihydrochloride (13)

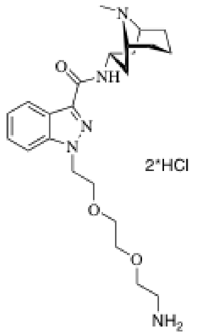

M. F. : $C_{23}H_{37}Cl_2N_5O_3$; M. W. : 502.48

tert-butyl (2-(2-(2-(3-(9-methyl-9-azabicyclo-[3.3.1]non-an-3-yl)carbamoyl)-1*H*-indazol-1-yl)ethoxy)ethoxy)-ethyl)carbamate (0.157 g, 0.295 mmol) was dissolved in dioxane (1.5 ml). HCl 4M in dioxane (1.47 ml, 5.9 mmol, 20 eq.) was then added. The mixture was stirred at RT for 4 h, the solvent was removed under high vacuum and co-evaporated 3 times with CH_2Cl_2 and washed with Et_2O. The product **13**•2 HCl (0.147 g, 0.292 mmol, 99% yield) was obtained as a white solid: IR (film): 3375, 2870, 2360, 2340, 1645, 1540, 1480, 1435, 1350, 1310, 1255, 1185, 1120 cm^{-1}. ^{1}H-NMR (300 MHz, CD_3OD): δ 8.19 (d, *J* = 8.2 Hz, 1H, indaz.) ; 7.69 (d, *J* = 8.6 Hz, 1H, indaz.) ; 7.46 (dd, *J* = 15.1, 0.6 Hz, 1H, indaz.) ; 7.29 (dd, *J* = 15.1, 0.6 Hz, 1H, indaz.) ; 4.79 – 4.62 (m, 3H) ; 4.01 (t, *J* = 5.2 Hz, 2H) ; 3.98 (t, *J* = 5.2 Hz, 2H) ; 2.53 (s, 3H) ; 2.46 (m, 2H) ; 2.24 – 1.93 (m, 2H) ; 1.70 – 1.51 (m, 3H) ; 1.43 (s, 9H) ; 1.16 (d, *J* = 12.7 Hz, 2H). ^{13}C-NMR (75 MHz, CD_3OD): δ 164.3 ; 143.1 ; 138.2 ; 133.7 ; 133.1 ; 133 ; 130.1 ; 129.9 ; 127.9 ; 123.9 ; 123.8 ; 122.8 ; 111.6 ; 72.5 ; 71.6 ; 71.4 ; 71.2 ; 70.8 ; 68.1 ; 67.9 ; 67.8 ; 55.2 ; 50.5 ; 49.9 ; 49.6 ; 49.3 ; 49.0 ; 48.7 ; 48.4 ; 48.2 ; 43.9 ; 40.6 ; 39.3 ; 38.5 ; 32.1 ; 24.1 ; 12.6. MS (ESI): 430 [M–2HCl+H]$^+$. HRMS-ESI: [M–2HCl+H, $C_{23}H_{36}N_5O_3$]: calculated 430.2813, found 430.2816.

1-(2-(2-(2-(7-(Diethylamino)-2-oxo-2*H*-chromene-3-carboxamido) ethoxy) ethoxy) ethyl)-*N*-(9-methyl-9-azabicyclo[3.3.1]nonan-3-yl)-1*H*-indazole-3-carboxamide (20)

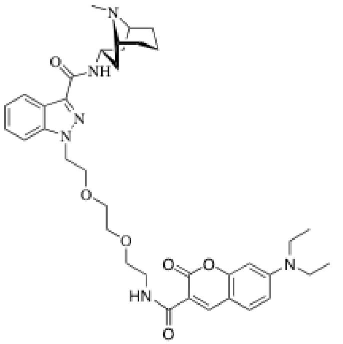

M. F. : $C_{37}H_{48}N_6O_6$; M. W. : 672.81

Following procedure I: coumarin carboxylic acid (0.02 g, 0.076 mmol, 1 eq.), DCC (0.02 g, 0.098 mmol, 1.3 eq.) and HOBt (0.01 g, 0.076 mmol, 1 eq.) were mixed in a two-neck round bottom flask in DMF/CH_2Cl_2. A solution of amine **13**•2 HCl (0.042 g, 0.084 mmol, 1.1 eq.) mixed with Et_3N (20 µl, 0.1 mmol, 2 eq.) in dry CH_2Cl_2 was then added drop wise. After work-up, the combined organic layers were dried over Na_2SO_4, filtered and concentrated to yield the crude product. The crude product was further purified by flash chromatography on silica gel (CH_2Cl_2/MeOH/Et_3N, 96:3:1). The product was then washed with water (4 ml), $NaHCO_3$ sat. (3 ml) and extracted with $CHCl_3$ (3×15 ml) to remove triethylamine impurities and to give fluorescent ligand **20** (0.036 g, 0.053 mmol, 70% yield) as a yellow solid: R_f = 0.74 (CH_2Cl_2/MeOH, 7:3). IR (neat): 2920, 2360, 1700, 1650, 1615, 1580, 1505, 1415, 1350, 1225, 1185, 1130, 855, 820, 790, 775, 750 cm^{-1}. ^{1}H-NMR (300 MHz, CD_3OD): δ 8.54 (s, 1H, coumarin) ; 8.14 (d, *J* = 8.2 Hz, 1H, indazole) ; 7.63 (d, *J* = 8.6 Hz, 1H, coum.) ; 7.46 (d, *J* = 9.0 Hz, 1H, indaz.) ; 7.37 (dd, *J* = 15.1, 1.0 Hz, 1H, indaz.) ; 7.22 (dd, *J* = 15.1, 1.0 Hz, 1H, indaz.) ; 6.76 (dd, *J* = 9.0, 2.4 Hz, 1H, coum.) ; 6.47 (d, *J* = 2.2 Hz, 1H, coum.) ; 4.62 (t, *J* = 5.3 Hz, 2H) ; 4.53 (tt, *J* = 11.6, 6.6 Hz, 1H) ; 3.99 (t, *J* = 5.3 Hz, 2H) ; 3.62 – 3.37 (m, 13H) ; 3.10 (d, *J* = 10.7 Hz, 2H) ; 2.52 (s, 3H, -NC*H*$_3$) ; 2.49 – 2.35 (m, 2H) ; 2.20 – 1.87 (m, 3H) ; 1.64 – 1.41 (m, 4H) ; 1.21 (t, *J* = 7.1 Hz, 6H, -N(CH_2C*H*$_3$)$_2$

coum.). ^{13}C-NMR (75 MHz, CD_3OD): δ 165.2 ; 164.2 ; 163.8 ; 159 ; 154.5 ; 149.2 ; 142.9 ; 138.6 ; 132.6 ; 127.7 ; 123.9 ; 123.5 ; 122.9 ; 111.6 ; 111.5 ; 110.1 ; 109.4 ; 97.2 ; 79.5 ; 71.8 ; 71.5 ; 70.9 ; 70.5 ; 52.9 ; 50.6 ; 47.2 ; 45.9 ; 41.4 ; 40.6 ; 40.5 ; 33.2 ; 30.7 ; 25.7 ; 14.9 ; 12.7 ; 10.5. MS (ESI): 673 $[M+H]^+$. HRMS-ESI: [M+H, $C_{37}H_{49}N_6O_6$] : calculated 673.3708, found 673.3714. UV-Vis/Fluo (Phosphate buffer pH 7): $\lambda_{max\ abs}$ 431 nm, $\lambda_{max\ em}$ 480 nm. Purity UPLC 99%, t_R = 2.12 min.

2,2,2-Trifluoroacetic acid salt with 2-(6-hydroxy-3-oxo-3*H*-xanthen-9-yl)-5-(3-(2-(2-(2-(3-((9-methyl-9-azabicyclo[3.3.1]nonan-3-yl)carbamoyl)-1*H*-indazol-1-yl)ethoxy)ethoxy)ethyl)thioureido)benzoic acid (21)

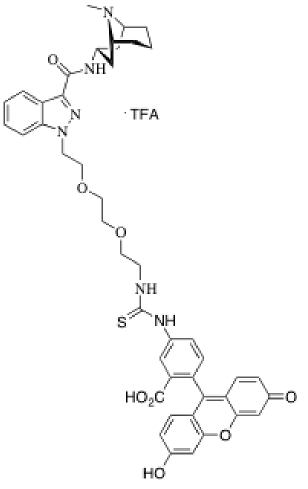

M. F. : $C_{46}H_{47}F_3N_6O_{10}S$; M. W. : 932.96

To a stirred solution of amine **13**•2 HCl (0.042 g, 0.084 mmol, 1 eq.) in DMF (1 ml) was added Et_3N (60 µl, 0.418 mmol, 5 eq.) and fluorescein isothiocyanate (0.036 g, 0.092 mmol, 1.1 eq.). The mixture was stirred for 5.5 h at room temperature. The solvent was removed under high vacuum. The crude product was purified by prep-LC using a gradient of 100% A (100% water with 0.1% of trifluoroacetic acid) to 100% D (40% water with 60% acetonitrile and 0.1% trifluoroacetic acid). The product had a retention time of 21 min. The fractions collected were dried by lyophilization to give **21**•TFA as an orange solid (0.015 g, 0.0161 mmol, 19% yield): IR (neat): 2950, 1670, 1535, 1275, 1175, 1115, 840 cm^{-1}. ^{1}H-NMR (300 MHz, CD_3OD): δ 8.17 (d, *J* = 8.2 Hz, 1H, indaz.) ; 8.13 (s, 1H, fluoresc.) ; 7.70 (d, *J* = 8.6 Hz, 1H, indaz.) ; 7.67 (s, 1H, fluoresc.) ; 7.46 (pseudo-t, *J* = 7.9 Hz, 1H, indaz.) ; 7.27 (pseudo-t, *J* = 7.6 Hz, 1H, indaz.) ; 7.16 (d, *J* = 8.2 Hz, 1H, fluoresc.) ; 6.78 – 6.66 (m, 4H, fluoresc.) ; 6.56 (d, *J* = 9.6 Hz, 2H, fluoresc.) ; 4.71 – 3.98 (m, 3H) ; 4.00 (t, *J* = 5.3 Hz, 2H) ; 3.82 – 3.46 (m, 10H) ; 2.96 (s, 3H) ; 2.66 – 2.43 (m, 2H) ; 2.19 (d, *J* = 6.6 Hz, 2H) ; 2.06 – 1.85 (m, 3H) ; 1.76 – 1.45 (m, 3H) ; 1.31 (d, *J* = 11.2 Hz, 1H). ^{13}C-NMR (75 MHz, CD_3OD): δ 187.1 ; 171.1 ; 164.4 ; 161.4 ; 154.2 ; 143.0 ; 138.1 ; 130.3 ; 128.9 ; 128.0 ; 125.7 ; 124.0 ; 122.8 ; 113.6 ; 111.6 ; 111.5 ; 103.5 ; 72.1 ; 71.7 ; 71.5 ; 71.4 ; 70.8 ; 67.9 ; 67.8 ; 56.0 ; 55.3 ; 45.4 ; 39.1 ; 38.3 ; 32.8 ; 32.1 ; 30.5 ; 24.1 ; 12.5 ; 9.2. MS (ESI): 819 $[M\text{–}TFA+H]^+$. HRMS-ESI: [M–TFA+H, $C_{44}H_{47}N_6O_8S$]: calculated 819.3171, found 819.3186. UV-Vis/Fluo (Phosphate buffer pH 7): $\lambda_{max\ abs}$ 498 nm, $\lambda_{max\ em}$ 524 nm. Purity UPLC 94%, t_R = 1.34 min.

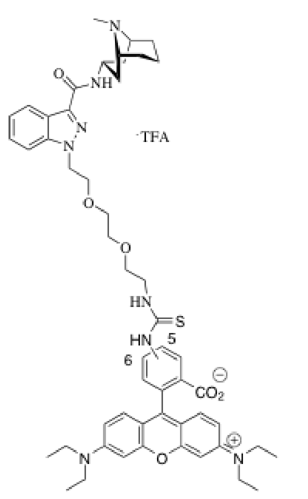

M. F. : $C_{54}H_{65}F_3N_8O_8S$; M. W. : 1043.2

2-(6-(Diethylamino)-3-(diethyliminio)-3*H*-xanthen-9-yl)benzoate compound with *N*-(9-methyl-9-azabicyclo[3.3.1]nonan-3-yl)-1-(3-thioxo-7,10-dioxa-2,4-diazadodecan-12-yl)-1*H*-indazole-3-carboxamide trifluoroacetic salt (22)

To a stirred solution of amine **13**•2 HCl (0.025 g, 0.049 mmol, 1 eq.) in DMF (0.5 ml) was added Et_3N (35 µl, 0.248 mmol, 5 eq.) and rhodamine B isothiocyanate (mixture of 5- and 6-isomers) (0.029 g, 0.055 mmol, 1.1 eq.). The mixture was stirred at room temperature. The reaction was followed by TLC and after showing completion, the solvent was

removed under high vacuum. The crude product was purified by prep-LC using a gradient of 100% A (100% water with 0.1% of trifluoroacetic acid) to 100% D (40% water with 60% acetonitrile and 0.1% trifluoroacetic acid). The product had a retention time of 21 min. The fractions collected were dried by lyophilization to give **22•**TFA as a purple solid (0.011 g, 0.011 mmol, 23% yield). The product was a mixture of 5- and 6-rhodamine isomers: IR (neat): 2920, 1590, 1125, 670 cm^{-1}. ^{1}H-NMR (400 MHz, CD_3OD): δ 8.45 (d, *J* = 2.2 Hz, 1H) ; 8.21 (d, *J* = 8.3 Hz, 1H) ; 8.10 – 7.99 (m, 1H) ; 7.70 (d, *J* = 8.6 Hz, 1H) ; 7.46 (dd, *J* = 11.4, 4.0 Hz, 2H) ; 7.32 (pseudo-t, *J* = 4.8 Hz, 1H) ; 7.30 (d, *J* = 2.7 Hz, 1H) ; 7.28 – 7.18 (m, 4H) ; 7.10 (d, *J* = 7.4 Hz, 1H) ; 7.05 (dd, *J* = 9.5, 2.6 Hz, 1H) ; 7.01 – 6.91 (m, 5H) ; 4.73 – 4.52 (m, 3H) ; 4.01 (t, *J* = 5.3 Hz, 2H) ; 3.81 – 3.47 (m, xH) ; 2.97 (s, 3H) ; 2.68 – 2.44 (m, 2H) ; 2.31 – 2.13 (m, 4H) ; 2.09 – 1.82 (m, 4H) ; 1.78 – 1.50 (m, 5H) ; 1.31 (t, *J* = 6.9 Hz, xH) ; 0.91 (t, *J* = 6.8 Hz, 2H). HSQC ^{13}C-NMR (75 MHz, CD_3OD): δ 173.4 ; 131 ; 130.5 ; 126.5 ; 125.0 ; 122.4 ; 121.2 ; 114 ; 113.6 ; 110.6 ; 95.9 ; 70.3 ; 69.6 ; 54.1 ; 49.2 ; 48.0 ; 45.8 ; 45.4 ; 37.0 ; 29.3 ; 11.5. MS (ESI): 929 $[M–TFA+H]^{+}$. HRMS-ESI: [M–TFA+H, $C_{52}H_{65}N_8O_6S$]: calculated 929.4742, found 929.4749. UV-Vis/Fluo (Phosphate buffer pH 7): $\lambda_{max\ abs}$ 557 nm, $\lambda_{max\ em}$ 584 nm. Purity UPLC 92%, t_R = 2.03 min.

(3) Synthesis of C7-linked granisetron conjugates

***tert*-butyl (2-((1-methyl-3-((9-methyl-9-azabicyclo[3.3.1]nonan-3-yl) carbamoyl)-1*H*-indazol-7-yl)oxy)ethyl)carbamate (33)**

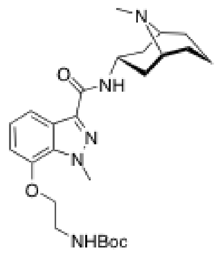

M. F. : $C_{25}H_{37}N_5O_4$; M. W. : 471.59

Following general procedure II: binding of linker to the granisetron core.

To a stirred solution of alcohol **31** (0.064 g, 0.194 mmol, 1 eq.) in DMF (2 ml), *tert*-butyl (2-bromoethyl)carbamate (0.069 g, 0.31 mmol, 1.6 eq.) was added, followed by potassium carbonate (0.048 g, 0.348 mmol, 1.8 eq.) and *n*-Bu_4NI (0.007 g, 0.019 mmol, 10 mol%) at room temperature. After stirring overnight at 60°C, the reaction mixture was partitioned between ethylacetate and water, the organic layer was washed with saturated aqueous NH_4Cl, saturated aqueous $NaHCO_3$ and brine, dried over $MgSO_4$ and filtered. The solvent was removed *in vacuo*. The crude product was purified on Alox basic (CH_2Cl_2/MeOH, 9.9:0.1 then 9:1) to give **33** as a white solid (0.058 g, 0.123 mmol, 64% yield). R_f = 0.8 (CH_2Cl_2/MeOH, 9:1) on Alox TLC. IR (neat): 3300, 2935, 2360, 2160, 1705, 1650, 1580, 1530, 1505, 1450, 1390, 1360, 1265, 1225, 1155, 1115, 1080, 1025, 1005, 940 cm^{-1}. ^{1}H-NMR (300 MHz, $CDCl_3$): δ 7.93 (d, *J* = 8.2 Hz, 1H) ; 7.08 (pseudo-t, *J* = 7.9 Hz, 1H) ; 6.75 (d, *J* = 8.3 Hz, 1H, N*H*, granatane) ; 6.66 (d, *J* = 7.6 Hz, 1H) ; 4.93 (s br, 1H, -N*H*Boc) ; 4.64 – 4.44 (m, 1H) ; 4.3 (s, 3H) ; 4.15 (t, *J* = 5.2 Hz, 2H) ; 3.65 (d, *J* = 5.1 Hz, 2H) ; 3.1 (d, *J* = 11.6, 2H) ; 2.6 – 2.43 (m, 5H) ; 2.31 – 2.13 (m, 1H) ; 2.05 – 1.81 (m, 3H) ; 1.45 (s, 9H) ; 1.4 – 1.3 (m, 3H) ; 1.06 (d, *J* = 9.9 Hz, 2H). ^{13}C-NMR (75 MHz, $CDCl_3$): δ 162.1 ; 155.9 ; 145.1 ; 137.5 ; 132.9 ; 125.3 ; 123.2 ; 115.4 ; 106.5 ; 67.7 ; 51.5 ; 49.1 ; 40.7 ; 39.6 ; 33.9 ; 33.1 ; 29.8 ; 28.5 ; 25.8 ; 24.9 ; 14.4. MS (ESI): 472 $[M+H]^{+}$. ESI-HRMS: [M+H, $C_{25}H_{38}N_5O_4$]: calculated 472.2918, found 472.2903.

7-(2-aminoethoxy)-1-methyl-*N*-(9-methyl-9-azabicyclo[3.3.1]nonan-3-yl)-1*H*-indazole-3-carboxamide dihydrochloride

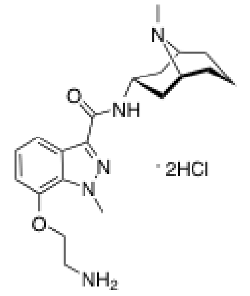

M. F. : $C_{20}H_{31}Cl_2N_5O_2$; M. W. : 444.4

Compound **33** (0.058 g, 0.123 mmol) was dissolved in dioxane (1.2 ml). HCl 4M in dioxane (0.615 ml, 2.46 mmol, 20 eq.) was then added. The mixture was left stirring at room temperature for 5 h, the solvent was removed under high vacuum and co-evaporated 3 times with CH_2Cl_2. The product was finally washed with Et_2O. Product, amine dihydrochloride salt (0.051 g, 0.114 mmol, 93% yield) was obtained as a white solid. IR (neat): 3330, 2930, 1620, 1540, 1505, 1395, 1265, 1115, 1025, 970, 820, 795, 750, 735 cm^{-1}. ^{1}H-NMR (300 MHz, CD_3OD): δ 7.80 (dd, *J* = 8.1, 2.3 Hz, 1H) ; 7.21 – 7.08 (m, 1H) ; 6.95 (dd, *J* = 7.5, 3.7 Hz, 1H) ; 4.69 (tt, *J* = 18.5, 6.2 Hz, 1H) ; 4.53 – 4.43 (m, 2H) ; 4.39 (s, 3H) ; 3.79 – 3.70 (m, 2H) ; 3.58 – 3.51 (m, 2H) ; 2.97 (s, 3H) ; 2.67 – 2.52 (m, 2H) ; 2.27 – 2.17 (m, 3H) ; 2.05 – 1.91 (m, 2H) ; 1.90 – 1.78 (m, 1H) ; 1.59 (d, *J* = 5.4 Hz, 2H). ^{13}C-NMR (75 MHz, CD_3OD): δ 164.3 ; 145.9 ; 139.6 ; 137.4 ; 133.7 ; 126.6 ; 124.7 ; 115.8 ; 108.2 ; 66.2 ; 55.8 ; 55.2 ; 43.6 ; 42.0 ; 40.5 ; 40.0 ; 39.1 ; 38.4 ; 34.6 ; 32.1 ; 31.3 ; 27.8 ; 26.6 ; 26.0 ; 24.0 ; 12.4. MS (ESI): 372 $[M–2HCl+H]^+$. ESI-HRMS: [M–2HCl+H, $C_{20}H_{30}N_5O_2$] : calculated 372.2394, found 372.2378.

7-(2-(7-(diethylamino)-2-oxo-2H-chromene-3-carboxamido)ethoxy)-1-methyl-N-(9-methyl-9-azabicyclo[3.3.1]nonan-3-yl)-1H-indazole-3-carboxamide (36)

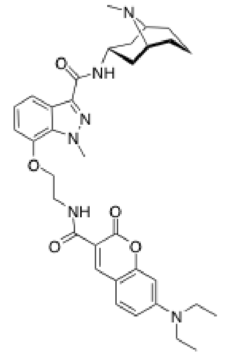

M. F. : $C_{34}H_{42}N_6O_5$; M. W. : 614.73

Following procedure I: coumarin carboxylic acid (0.019 g, 0.072 mmol, 1 eq.), DCC (0.019 g, 0.093 mmol, 1.3 eq.) and HOBt (0.01 g, 0.072 mmol, 1 eq.) were mixed in a two-neck round bottom flask in DMF/CH_2Cl_2. A solution of above amine•2HCl (0.035 g, 0.079 mmol, 1.1 eq.) mixed with Et_3N (20 µl, 0.143 mmol, 2 eq.) in dry CH_2Cl_2 was then added dropwise. After work-up, the combined organic layers were dried over Na_2SO_4, filtered and concentrated to yield the crude product. The crude product was further purified by flash chromatography on silica gel (CH_2Cl_2/MeOH/Et_3N, 96:3:1), the solvents were removed under reduced pressure. The product was then washed with water (4 ml), $NaHCO_3$ sat. (3 ml) and extracted with $CHCl_3$ (3×15 ml) to remove the triethylamine salt and to give fluorescent ligand **36** (0.039 g, 0.063 mmol, 88% yield) as a yellow solid. R_f = 0.74 (CH_2Cl_2/MeOH, 7:3). IR (neat): 3330, 2920, 2360, 1700, 1650, 1615, 1580, 1505, 1455, 1415, 1350, 1255, 1225, 1190, 1135, 1080, 820, 790, 730 cm^{-1}. ^{1}H-NMR (300 MHz, $CDCl_3$): δ 9.26 (t, *J* = 5.6 Hz, 1H, amide) ; 8.71 (s, 1H, coum.) ; 7.92 (d, *J* = 8.1 Hz, 1H, indaz.) ; 7.43 (d, *J* = 9.0 Hz, 1H) ; 7.09 (t, *J* = 7.9 Hz, 1H) ; 6.77 (d, *J* = 8.4 Hz, 1H) ; 6.70 (d, *J* = 7.6 Hz, 1H, indaz.) ; 6.64 (dd, *J* = 9.0, 2.4 Hz, 1H, indaz.) ; 6.48 (d, *J* = 2.2 Hz, 1H, coum.) ; 4.53 (tt, *J* = 11.6, 6.6 Hz, 1H) ; 4.38 (s, 3H) ; 4.29 (t, *J* = 5.2 Hz, 2H) ; 3.97 (dd, *J* = 10.7, 5.3 Hz, 2H) ; 3.44 (q, *J* = 7.1 Hz, 4H) ; 3.10 (d, *J* = 10.5 Hz, 2H) ; 2.58 – 2.43 (m, 5H) ; 2.20 – 1.87 (m, 3H) ; 1.57 – 1.48 (m, 1H) ; 1.46 – 1.32 (m, 2H) ; 1.21 (t, *J* = 7.1 Hz, 6H, $-N(CH_2CH_3)_2$ coum.) ; 1.15 – 0.98 (m, 2H). ^{13}C-NMR (75 MHz, $CDCl_3$): δ 163.6 ; 162.8 ; 162.2 ; 157.7 ; 152.8 ; 148.3 ; 145.2 ; 137.2 ; 132.8 ; 131.3 ; 125.3 ; 123.2 ; 115.3 ; 110.1 ; 110.0 ; 108.5 ; 106.2 ; 96.5 ; 67.1 ; 51.5 ; 45.2 ;

40.8 ; 40.7 ; 40.0 ; 39.0 ; 33.0 ; 29.8 ; 25.1 ; 14.3 ; 14.2 ; 12.4. MS (ESI): 615 $[M+H]^+$. ESI-HRMS: [M+H, $C_{34}H_{43}N_6O_5$] : calculated 615.3289, found 615.3275. UV-Vis/Fluo (Phosphate buffer pH 7): $\lambda_{max\ abs}$ 430 nm, $\lambda_{max\ em}$ 479 nm. Purity UPLC 96%, t_R = 1.62 min.

***tert*-Butyl (3-((1-methyl-3-((9-methyl-9-azabicyclo[3.3.1]nonan-3-yl)carbamoyl)-1*H*-indazol-7-yl)oxy)propyl)carbamate (34)**

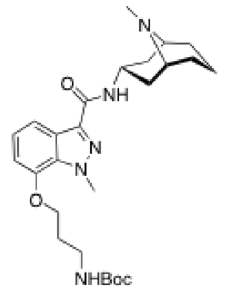

M. F. : $C_{26}H_{39}N_5O_4$; M. W. : 485.61

Following general procedure II: alcohol **31** (0.145 g, 0.442 mmol) was stirred in DMF (4.4 ml), linker **8** (0.168 g, 0.71 mmol, 1.6 eq.) was added, followed by K_2CO_3 (0.110 g, 0.795 mmol, 1.8 eq.) and *n*-Bu_4NI (0.016 g, 0.044 mmol, 10 mol%). The solvent was removed *in vacuo*. The product **34** was a white solid (0.2 g, 0.412 mmol, 93% yield) that was used in the next step without further purification: IR (film): 2920, 2360, 1700, 1535, 1260, 1170 cm^{-1}. ^{1}H-NMR (300 MHz, $CDCl_3$): δ 7.93 (d, *J* = 8.1 Hz, 1H, arom.) ; 7.10 (pseudo-t, *J* = 7.9 Hz, 1H, arom.) ; 6.77 – 6.71 (m, 1H, -N*H*, bicyclic amine) ; 6.70 (d, *J* = 7.6 Hz, 1H, arom.) ; 4.73 (broad s, 1H, -N*H*Boc) ; 4.54 (tt, *J* = 12.6, 6.4 Hz, 1H) ; 4.32 (s, 3H, -NC*H*$_3$) ; 4.17 (t, *J* = 6.0 Hz, 2H) ; 3.38 (t, *J* = 5.7 Hz, 2H) ; 3.09 (d, *J* = 11.0 Hz, 2H) ; 2.61 – 2.41 (m, 5H) ; 2.16 – 2.02 (m, 2H) ; 1.96 (dd, *J* = 10.8, 3.5 Hz, 3H) ; 1.43 (s, 9H, Boc) ; 1.41 – 1.29 (m, 3H) ; 1.11 – 0.81 (m, 2H). ^{13}C-NMR (75 MHz, $CDCl_3$): δ 162.1 ; 155.7 ; 145.1 ; 137.1 ; 132.6 ; 125.1 ; 123.1 ; 114.6 ; 106.4 ; 66.1 ; 51.5 ; 40.2 ; 39.7 ; 32.1 ; 29.8 ; 28.4 ; 25.1 ; 13.8. MS (ESI): 486 $[M+H]^+$. HRMS-ESI: [M+H, $C_{26}H_{40}N_5O_4$]: calculated 486.3075, found 486.3068.

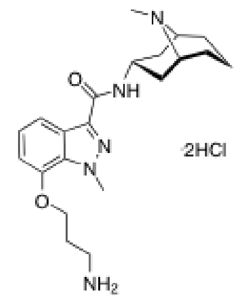

M. F. : $C_{21}H_{33}Cl_2N_5O_2$; M. W. : 458.42

7-(3-Aminopropoxy)-1-methyl-*N*-(9-methyl-9-azabicyclo[3.3.1]nonan-3-yl)-1*H*-indazole-3-carboxamide dihydrochloride

Compound **34** (0.2 g, 0.42 mmol) was dissolved in dioxane (4 ml). HCl 4M in dioxane (2 ml, 8 mmol, 20 eq.) was then added. The mixture was stirred at room temperature for 4 h. The solvent was removed under high vacuum and co-evaporated 3 times with CH_2Cl_2. The product was washed with Et_2O. This gave the title compound (0.184 g, 0.402 mmol, 98% yield) as a white solid: IR (neat): 3370, 2950, 2495, 2080, 1645, 1585, 1550, 1485, 1445, 1395, 1350, 1330, 1305, 1265, 1140, 1115, 1070, 1030, 970, 880 cm^{-1}. ^{1}H-NMR (300 MHz, CD_3OD):δ 7.72 (d, *J* = 8.1 Hz, 1H, arom.) ; 7.17 – 7.07 (m, 1H, arom.) ; 6.84 (d, *J* = 7.6 Hz, 1H, arom.) ; 4.72 (tt, *J* = 12.1, 6.2 Hz, 1H) ; 4.33 (s, 3H, -NCH3) ; 4.27 (t, *J* = 5.9 Hz, 2H) ; 3.77 (d, *J* = 12.2 Hz, 2H) ; 3.28 – 3.21 (m, 2H) ; 2.97 (s, 3H) ; 2.76 – 2.50 (m, 2H) ; 2.37 – 2.08 (m, 3H) ; 2.04 – 1.89 (m, 3H) ; 1.74 – 1.52 (m, 2H) ; 1.48 – 1.22 (m, 2H). ^{13}C-NMR (75 MHz, CD_3OD): δ 164.3 ; 146.5 ; 137.5 ; 133.8 ; 129.9 ; 126.2 ; 124.7 ; 114.9 ; 107.8 ; 66.3 ; 55.1 ; 42.2 ; 40.4 ; 39.9 ; 39.1 ; 38.5 ; 32.1 ; 28.7 ; 28.3 ; 24.1 ; 14.4 ; 12.5. MS (ESI): 386 $[M–2HCl+H]^+$. HRMS-ESI: [M–2HCl+H, $C_{21}H_{32}N_5O_2$]: calculated 386.2556, found 386.2562.

7-(3-(7-(Diethylamino)-2-oxo-2*H*-chromene-3-carboxamido)propoxy)-1-methyl-N-(9-methyl-9-azabicyclo[3.3.1]nonan-3-yl)-1*H*-indazole-3-carboxamide (37)

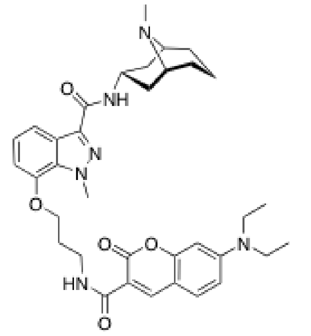

M. F. : $C_{35}H_{44}N_6O_5$; M. W. : 628.76

Following procedure I: coumarin carboxylic acid (0.023 g, 0.089 mmol, 1 eq.), DCC (0.024 g, 0.116 mmol, 1.3 eq.) and HOBt (0.012 g, 0.089 mmol, 1 eq.) were mixed in DMF/CH_2Cl_2. A solution of the amine dihydrochloride salt from the previous step (0.045 g, 0.098 mmol, 1.1 eq.) was mixed with Et_3N (25 µl, 0.178 mmol, 2 eq.) in dry CH_2Cl_2 and then added drop-wise. After work-up, the combined organic layers were dried over Na_2SO_4, filtered and concentrated to yield the crude product. The crude product was further purified by flash chromatography on silica gel (CH_2Cl_2/MeOH/Et_3N, 96:3:1), the solvents were removed under reduced pressure. The product was then washed with water (4 ml), $NaHCO_3$ sat. (3 ml) and extracted with $CHCl_3$ (3×15 ml) to remove triethylamine impurities and gave fluorescent ligand **37** (0.048 g, 0.076 mmol, 86% yield) as a yellow solid: R_f = 0.63 (CH_2Cl_2/MeOH, 7:3). IR (neat): 3340, 2920, 2025, 1695, 1650, 1615, 1580, 1505, 1415, 1350, 1255, 1225, 1185, 1130, 1075, 820, 790, 730 cm^{-1}. ^{1}H-NMR (300 MHz, $CDCl_3$): δ 8.96 (t, *J* = 5.5 Hz, 1H, amide) ; 8.69 (s, 1H, coumarin) ; 7.91 (d, *J* = 8.2 Hz, 1H, indazole) ; 7.42 (d, *J* = 9.0 Hz, 1H, indaz.) ; 7.14 – 6.99 (m, 1H, coum.) ; 6.73 (d, *J* = 9.0 Hz, 1H, indaz.) ; 6.64 (dd, *J* = 9.0, 2.4 Hz, 1H, indaz.) ; 6.49 (d, *J* = 2.4 Hz, 1H, indaz.) ; 4.55 (tt, *J* = 12.1, 6.2 Hz, 1H) ; 4.35 (s, 3H) ; 4.21 (t, *J* = 6.1 Hz, 2H) ; 3.69 (t, *J* = 7.0 Hz, 2H) ; 3.45 (q, *J* = 7.1 Hz, 4H) ; 3.08 (d, *J* = 10.5 Hz, 2H) ; 2.60 – 2.41 (m, 5H) ; 2.24 (dd, *J* = 13.1, 6.5 Hz, 2H) ; 2.06 – 1.84 (m, 2H) ; 1.77 – 1.46 (m, 2H) ; 1.46 – 1.29 (m, 2H) ; 1.23 (t, *J* = 7.1, 6H) ; 1.17 – 0.96 (m, 2H). ^{13}C-NMR (75 MHz, $CDCl_3$): δ 163.5 ; 163.4 ; 162.9 ; 162.9 ; 162.1 ; 162.0 ; 157.8 ; 152.7 ; 148.2 ; 145.4 ; 137.3 ; 132.9 ; 131.2 ; 125.3 ; 123.4 ; 114.9 ; 110.1 ; 108.4 ; 106.3 ; 96.7 ; 77.6 ; 77.4 ; 77.2 ; 76.7 ; 65.9 ; 51.5 ; 49.0 ; 48.9 ; 45.2 ; 40.7 ; 40.6 ; 39.8 ; 36.8 ; 36.7 ; 34.0 ; 33.0 ; 29.8 ; 29.5 ; 25.7 ; 25.1 ; 25.0 ; 14.4 ; 12.5. MS (ESI): 629 $[M+H]^+$. HRMS-ESI: [M+H, $C_{35}H_{45}N_6O_5$]: calculated 629.3446, found 629.3423. UV-Vis/Fluo (Phosphate buffer pH 7): $\lambda_{max\ abs}$ 430 nm, $\lambda_{max\ em}$ 478 nm. Purity UPLC 91%, t_R = 2.09 min.

***tert*-Butyl (2-(2-(2-((1-methyl-3-(9-methyl-9-azabicyclo[3.3.1]nonan-3-yl)carbamoyl)-1*H*-indazol-7-yl)oxy)ethoxy)ethoxy)ethyl)carbamate (35)**

Following general procedure II: binding of linker to the granisetron core.

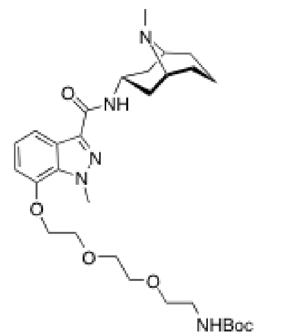

M. F. : $C_{29}H_{45}N_5O_6$; M. W. : 559.69

To a stirred solution of alcohol **31** (0.106 g, 0.323 mmol, 1 eq.) in DMF (3 ml), linker **10** (0.161 g, 0.52 mmol, 1.6 eq.) was added, followed by K_2CO_3 (0.08 g, 0.58 mmol, 1.8 eq.) and *n*-Bu_4NI (0.012 g, 0.032 mmol, 10 mol%). After work-up, the crude product was purified on basic Alox (AcOEt followed by CH_2Cl_2/MeOH, 9:1) which yielded product **35** as a white solid (0.126 g, 0.323 mmol, 70% yield): R_f = 0.5 (CH_2Cl_2/MeOH, 9:1) on Alox TLC. IR (film): 2360, 2345 cm^{-1}. ^{1}H-NMR (300 MHz, CD_3OD): δ 7.74 (d, *J* = 8.2 Hz, 1H) ; 7.11 (pseudo-t, *J* = 7.9 Hz, 1H) ; 6.83 (d, *J* = 7.6 Hz, 1H) ; 4.56 (tt, *J* = 11.5,

5.8 Hz, 1H) ; 4.35 (s, 3H) ; 4.32 – 4.26 (m, 2H) ; 3.98 – 3.90 (m, 2H) ; 3.75 – 3.68 (m, 2H) ; 3.67 – 3.59 (m, 2H) ; 3.49 (t, *J* = 5.6 Hz, 2H) ; 3.20 (t, *J* = 5.6 Hz, 2H) ; 3.09 (d, *J* = 10.5 Hz, 2H) ; 2.51 (s, 3H) ; 2.49 – 2.34 (m, 2H) ; 2.17 – 1.92 (m, 3H) ; 1.62 – 1.46 (m, 3H) ; 1.39 (s, 9H) ; 1.12 (d, *J* = 12.5 Hz, 2H). ^{13}C-NMR (75 MHz, CD_3OD): δ 164.2 ; 147.0 ; 138 ; 134.2 ; 126.4 ; 124.6 ; 115.3 ; 108.1 ; 80.1 ; 72.4 ; 71.7 ; 71.4 ; 71.3 ; 71.1 ; 70.7 ; 69.2 ; 61.6 ; 52.8 ; 41.5 ; 41.3 ; 40.7 ; 40.3 ; 33.3 ; 31.4 ; 30.8 ; 28.8 ; 25.7 ; 14.9 ; 14.5. MS (ESI): 560 $[M+H]^+$. HRMS-ESI: [M+H, $C_{29}H_{46}N_5O_6$]: calculated 560.3443, found 560.3447.

7-(2-(2-(2-Aminoethoxy)ethoxy)ethoxy)-1-methyl-*N*-(9-methyl-9-azabicyclo[3.3.1]nonan-3-yl)-1*H*-indazole-3-carboxamide dihydrochloride

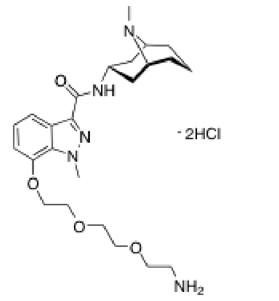

M. F. : $C_{24}H_{39}Cl_2N_5O_4$; M. W. : 532.5

Compound **35** (0.117 g, 0.21 mmol) was dissolved in dioxane (1 ml). HCl 4M in dioxane (1.05 ml, 4.2 mmol, 20 eq.) was then added. The mixture was stirred at room temperature for 2 h, the solvent was removed under high vacuum and co-evaporated 3 times with CH_2Cl_2. The product was washed with Et_2O and afforded the amine dihydrochloride salt (0.118 g, 0.402 mmol, quantitative yield) as a slightly brown solid: IR (film): 3400, 2920, 2360, 2165, 2030, 1650, 1540, 1260 cm^{-1}. ^{1}H-NMR (300 MHz, CD_3OD): δ 7.76 (d, *J* = 8.1 Hz, 1H) ; 7.16 (pseudo-t, *J* = 7.9 Hz, 1H) ; 6.90 (d, *J* = 7.6 Hz, 1H) ; 4.64 (tt, *J* = 12.2, 6.6 Hz, 1H) ; 4.38 (s, 3H) ; 4.37 – 4.30 (m, 2H) ; 4.06 – 3.93 (m, 2H) ; 3.85 – 3.68 (m, 9H) ; 3.15 – 3.07 (m, 2H) ; 2.98 (s, 3H) ; 2.76 – 2.52 (m, 2H) ; 2.30 – 2.15 (m, 3H) ; 2.07 – 1.92 (m, 2H) ; 1.76 – 1.54 (m, 3H). ^{13}C-NMR (75 MHz, CD_3OD): δ 164.4 ; 147.0 ; 137.5 ; 134.2 ; 126.4 ; 124.8 ; 115.1 ; 108.2 ; 71.7 ; 71.4 ; 70.7 ; 69.2 ; 68.1 ; 67.9 ; 56.0 ; 55.2 ; 54.8 ; 40.7 ; 40.2 ; 39.2 ; 38.4 ; 32.1 ; 31.2 ; 30.7 ; 27.9 ; 24.1 ; 12.6. MS (ESI): 460 $[M+H]^+$. HRMS-ESI: [M+H, $C_{24}H_{38}N_5O_4$]: calculated 460.2918, found 460.2922.

7-(2-(2-(2-(7-(Diethylamino)-2-oxo-2*H*-chromene-3-carboxamido)ethoxy)ethoxy)ethoxy)-1-methyl-*N*-(9-methyl-9-azabicyclo[3.3.1]nonan-3-yl)-1*H*-indazole-3-carboxamide (38)

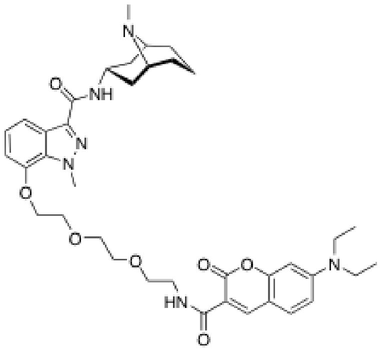

M. F. : $C_{38}H_{50}N_6O_7$; M. W. : 702.84

Following procedure I: coumarin carboxylic acid (0.013 g, 0.05 mmol, 1 eq.), DCC (0.014 g, 0.066 mmol, 1.3 eq.) and HOBt (0.007 g, 0.05 mmol, 1 eq.) were mixed in DMF/CH_2Cl_2. A solution of the amine dihydrochloride salt (0.03 g, 0.056 mmol, 1.1 eq.) from the previous step mixed with Et_3N (14 µl, 0.102 mmol, 2 eq.) in dry CH_2Cl_2 was then added drop-wise. After work-up, the combined organic layers were dried over Na_2SO_4, filtered and concentrated to yield the crude product. The crude product was further purified by flash chromatography on silica gel (CH_2Cl_2/MeOH/Et_3N, 96:3:1). The product was then washed with water (4 ml), $NaHCO_3$ sat. (3 ml), extracted with $CHCl_3$ (3×15 ml) to remove triethylamine impurities and afforded fluorescent ligand **38** (0.033 g, 0.047 mmol, 92% yield) as a yellow solid: R_f = 0.74 (CH_2Cl_2/MeOH, 7:3). IR (neat): 2920, 2360, 1700, 1650, 1615, 1580, 1505, 1415, 1350, 1260, 1225, 1185, 1130, 820, 795, 735 cm^{-1}. ^{1}H-NMR

(300 MHz, $CDCl_3$): δ 9.05 (s, 1H) ; 8.63 (s, 1H) ; 7.84 (d, *J* = 8.1 Hz, 1H) ; 7.34 (d, *J* = 9.0 Hz, 1H) ; 7.04 (pseudo-t, *J* = 7.9 Hz, 1H) ; 6.74 (d, *J* = 8.4 Hz, 1H) ; 6.68 (d, *J* = 7.6 Hz, 1H) ; 6.59 (dd, *J* = 9.0, 2.4 Hz, 1H) ; 6.38 (d, *J* = 2.4 Hz, 1H) ; 4.62 – 4.42 (m, 1H) ; 4.30 (s, 3H) ; 4.28 – 4.22 (m, 2H) ; 4.13 (s, 1H) ; 4.03 – 3.96 (m, 2H) ; 3.79 – 3.72 (m, 2H) ; 3.72 – 3.68 (m, 2H) ; 3.66 (s, 4H) ; 3.43 (q, *J* = 7.0 Hz, 6H) ; 3.09 (d, *J* = 10.5 Hz, 2H) ; 2.59 – 2.43 (m, 5H) ; 2.02 – 1.86 (m, 2H) ; 1.84 – 1.47 (m, 3H) ; 1.43 – 1.28 (m, 3H) ; 1.23 (t, *J* = 7.1 Hz, 6H). ^{13}C-NMR (75 MHz, $CDCl_3$): δ 163.4 ; 162.7 ; 162.1 ; 157.6 ; 152.6 ; 148.1 ; 145.4 ; 137.3 ; 133.0 ; 131.1 ; 125.2 ; 123.2 ; 115.0 ; 110.2 ; 110.0 ; 108.4 ; 106.7 ; 96.6 ; 71.1 ; 70.8 ; 70.0 ; 69.9 ; 67.9 ; 51.5 ; 46.2 ; 45.2 ; 40.8 ; 39.7 ; 33.2 ; 29.8 ; 25.0 ; 14.4 ; 12.6 ; 10.9. MS (ESI): 703 $[M+H]^+$. HRMS-ESI: [M+H, $C_{29}H_{46}N_5O_6$] : calculated 703.3814, found 703.3800. UV-Vis/Fluo (Phosphate buffer pH 7): $\lambda_{max\ abs}$ 437 nm, $\lambda_{max\ em}$ 484 nm. Purity UPLC 91%, t_R = 1.97 min.

3-(7-(2-(2-(2-(3-(3-carboxy-4-(6-hydroxy-3-oxo-3H-xanthen-9-yl)phenyl) thioureido)ethoxy)ethoxy)ethoxy)-1-methyl-1H-indazole-3-carboxamido)-9,9-dimethyl-9-azabicyclo[3.3.1]nonan-9-ium 2,2,2-trifluoroacetate (39)

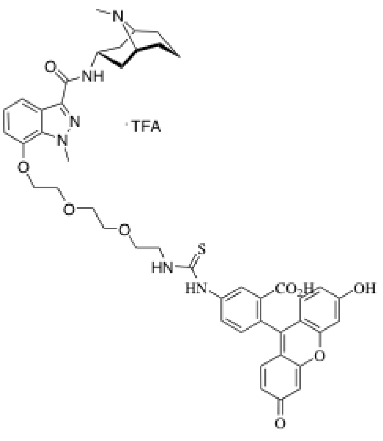

M. F. : $C_{47}H_{49}F_3N_6O_{11}S$; M. W. : 962.99

To a stirred solution of amine•2HCl (0.035 g, 0.066 mmol, 1 eq.) in DMF (0.5 ml) was added triethylamine (46 µl, 0.328 mmol, 5 eq.) and fluorescein isothiocyanate (0.028 g, 0.072 mmol, 1.1 eq.). The reaction was followed by TLC and showed completion after 5h at room temperature. The solvent was removed under high vacuum. The crude product was purified by prep-LC, the eluent used was a gradient of 50% A (100% water with 0.1% of trifluoroacetic acid) to 50% D (40% water with 60% acetonitrile and 0.1% trifluoroacetic acid) in 5 minutes then 30% A 70% D in 30 minutes. The product had a retention time of 9 min. The fractions collected were dried by lyophilization to give **39**•TFA as an orange solid (0.05 g, 0.052 mmol, 79% yield). IR (neat): 2950, 1675, 1585, 1540, 1455, 1260, 1175, 1115, 840, 795, 720 cm^{-1}. ^{1}H-NMR (300 MHz, CD_3OD): δ 8.24 (d, *J* = 1.7 Hz, 1H) ; 7.67 (d, *J* = 8.2 Hz, 1H) ; 7.59 (d, *J* = 4.8 Hz, 1H) ; 7.08 (dt, *J* = 8.0, 3.8 Hz, 2H) ; 6.82 (d, *J* = 7.6 Hz, 1H) ; 6.77 – 6.70 (m, 4H) ; 6.59 (dd, *J* = 8.8, 2.2 Hz, 2H) ; 4.55 (tt, *J* = 18.1, 12.0, 6.3 Hz, 1H) ; 4.34 (s, 3H) ; 4.33 – 4.25 (m, 2H) ; 4.01 – 3.92 (m, 2H) ; 3.75 (dd, *J* = 19.0, 3.7 Hz, 10H) ; 2.93 (s, 3H) ; 2.70 – 2.43 (m, 2H) ; 2.18 (d, *J* = 6.8 Hz, 2H) ; 2.01 – 1.84 (m, 3H) ; 1.69 – 1.44 (m, 3H). ^{13}C-NMR (75 MHz, CD_3OD): δ 170.8 ; 164.2 ; 161.6 ; 154.2 ; 149.5 ; 149.0 ; 148.2 ; 146.9 ; 146.9 ; 142.4 ; 139.2 ; 137.4 ; 134.2 ; 130.3 ; 128.8 ; 126.3 ; 125.6 ; 124.7 ; 115.0 ; 113.8 ; 111.7 ; 108.3 ; 103.5 ; 71.6 ; 71.4 ; 70.6 ; 69.3 ; 55.2 ; 45.5 ; 40.3 ; 39.1 ; 38.2 ; 32.1 ; 24.0 ; 12.4. MS (ESI): 849 $[M–TFA+H]^+$. ESI-HRMS: [M–TFA+H, $C_{45}H_{49}N_6O_9S$]: calculated 849.3276, found 849.3260. UV-Vis/Fluo (Phosphate buffer pH 7): $\lambda_{max\ abs}$ 498 nm, $\lambda_{max\ em}$ 519 nm. Purity UPLC 98%, t_R = 1.84 min.

Methyl 7-(diethylamino)-2-oxo-2*H*-chromene-3-carboxylate

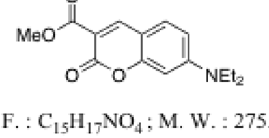

M. F. : $C_{15}H_{17}NO_4$; M. W. : 275.29

4-diethylamino-salicylaldehyde (0.3 g, 1.551 mmol) was dissolved in MeOH (15ml), dimethyl malonate was added

(0.18 ml, 1.551 mmol) followed by piperidine (0.153 ml, 1.551 mmol). The mixture was stirred for 4 h under N_2 at reflux. Progress of the reaction was followed by TLC. The solution was cooled to room temperature and water was added (30 ml). The aqueous layer was extracted with CH_2Cl_2 (3×30 ml), the pH of water was adjusted to 7, $NaHCO_3$ sat. (15 ml) was added and extracted again with CH_2Cl_2 (30 ml). The combined organic layers were dried over Na_2SO_4, filtered and concentrated under reduced pressure. The crude product was purified by flash chromatography on silica gel (CH_2Cl_2/MeOH/Et_3N, 96:3:1) to give the title compound as a dark yellow oil (0.404 g, 1.47 mmol, 95% yield): R_f = 0.37 (CH_2Cl_2/AcOEt, 9:1). IR (film): 2975, 2360, 1755, 1615, 1585, 1510, 1445, 1415, 1350, 1220, 1190, 1135, 800 cm^{-1}. ^1H-NMR (300 MHz, CD_3OD): δ 8.56 (s, 1H, arom.) ; 7.52 (d, J = 9.0 Hz, 1H, arom.) ; 6.80 (d, J = 9.0 Hz, 1H, arom.) ; 6.53 (s, 1H, arom.) ; 3.85 (s, 3H, -OMe) ; 3.54 (q, J = 7.1 Hz, 4H, -NEt_2) ; 1.24 (t, J = 7.1 Hz, 6H, -NEt_2). ^{13}C-NMR (75 MHz, CD_3OD): δ 165.7 ; 159.5 ; 154.9 ; 150.8 ; 144.9 ; 132.7 ; 111.2 ; 108.8 ; 107.9 ; 97 ; 54.8 ; 52.4 ; 45.8 ; 25.3 ; 12.7. MS (ESI): 276 $[M+H]^+$. HRMS-ESI: [M+H, $C_{15}H_{18}NO_4$]: calculated 276.1230, found 276.1239.

7-(Diethylamino)-2-oxo-2*H*-chromene-3-carboxylic acid

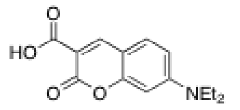

M. F. : $C_{14}H_{15}NO_4$; M. W. : 261.27

Methyl 7-(diethylamino)-2-oxo-2*H*-chromene-3-carboxylate (0.404 g, 1.47 mmol) was dissolved in 2M NaOH (12 ml). The reaction was stirred overnight at room temperature. 2M HCl was added until the solution reached pH 2. It was then extracted with CH_2Cl_2 (4×30 ml) and washed with water (20 ml). The combined organic layers were dried over Na_2SO_4, filtered and concentrated under reduced pressure to give the coumarin carboxylic acid product as an orange solid (0.216 g, 0.826 mmol, 56% yield): IR (film): 2920, 1730, 1660, 1600, 1575, 1510, 1450, 1400, 1355, 1315, 1265, 1185, 1130, 1080, 1005, 960, 790 cm^{-1}. ^1H-NMR (300 MHz, $CDCl_3$): δ 8.65 (s, 1H, arom.) ; 7.58 (d, J = 9.0 Hz, 1H, arom.) ; 6.88 (dd, J = 9.0, 2.5 Hz, 1H, arom.) ; 6.64 (s, 1 H, arom.) ; 3.55 (q, J = 7.2 Hz, 4H, $N(C\mathit{H}_2CH_3)_2$) ; 1.25 (t, J = 7.1 Hz, 6H, $N(CH_2C\mathit{H}_3)_2$). ^{13}C-NMR (75 MHz, CD_3OD): 164.8 (arom.) ; 158.3 (arom.) ; 154.1 (arom.) ; 150.7 (arom.) ; 132.3 (arom.) ; 111.1 (arom.) ; 108.7; 105.6; 97.2 (arom.) ; 45.5 ($N(\mathit{C}H_2CH_3)_2$) ; 12.5 ($N(CH_2\mathit{C}H_3)_2$). MS (ESI): 262 $[M+H]^+$. HRMS-ESI: [M+H, $C_{14}H_{16}NO_4$]: calculated 262.1074, found 262.1083.

(4) Fluorescence spectroscopy measurements

Absorption spectra were measured with a Varian Cary 100 Bio UV-visible Spectrophotometer, emission spectra were measured with a Varian Cary Eclipse fluorescence Spectrophotometer.

For measuring $\lambda_{max\ abs}$ and $\lambda_{max\ em}$ (Stoke's shift) and the extinction coefficient, the concentrations of the samples were in the 10^{-5} to 10^{-6} M range. A blank measurement of the solvent was done and its absorbance was subtracted from the sample. The equation used was:

$$\varepsilon = A/([c] \times l) \qquad [M^{-1}cm^{-1}]$$

where ε is the extinction coefficient, A is the absorbance of the sample, [c] its concentration and l the path length of the cuvette (1 cm).

The method of choice for the determination of fluorescence quantum yields was to relate the quantum yield of the sample to that of a reference standard.[S4] Measurements were done in MeOH (highest quality for analysis) and in pH 7 phosphate buffer solution at 20°C and 25°C depending on the standard used as reference. In order to avoid inner filter effects, the concentration of the samples was adjusted such that the absorbance was between 0.05 and 0.08 at and above the excitation wavelength. For all calculations the solvent background was subtracted from the sample. The equation used to relate these quantum yields is

$$\Phi_{Fx} = [(A_{std}(\lambda_{exc}) / A_x(\lambda_{exc})) \times (F_x/F_{std}) \times (n_x/n_{std})^2] \times \Phi_{Fstd},$$

where the subscript *x* refers to the unknown sample and the subscript *std* refers to the standard; Φ_F is the fluorescence quantum yield, *F* is the integrated fluorescence intensity, *A* is the absorbance at the excitation wavelength and *n* is the refractive index of the solvent. The standards were chosen such that the absorbance of the standard was overlapping with the absorbance of the sample. The reference standards used were quinine sulphate in 0.5M H_2SO_4 (Φ_F = 0.54)[S5], coumarin 6 in acetonitrile (Φ_F = 0.63)[S6], fluorescein in 0.1M NaOH (Φ_F = 0.95)[S7] and rhodamine B in ethanol (Φ_F = 0.7)[S8].

(5) Radioligand binding and fluorescence microscopy

Materials, cell culture maintenance, receptor expression and radioligand binding for the binding affinity measurements were described previously.[13]

For the fluorescence microscopy experiments COS-7 cells were transiently transfected with human 5-HT3A-myc cDNA (10 µg per transfection of 2 x 10^{-6} cells) by electroporation (400 V, ∞ Ω, 125 µF), and grown on coverslips for 24 hours. Cells were washed with HBS then incubated with 100 nM of each probe (diluted in HBS) for 1 hour at room temperature in the dark. In some experiments 10 µM unlabelled ondansetron (OND) was co-incubated with the probes. Excess probe was removed by washing four times in HBS, and coverslips were placed in an imaging chamber with 1 ml HBS. Cells were then imaged using a fluorescence microscope set to the appropriate emission/excitation wavelengths.

[S4] Fery-Forgues, S.; Lavabre, D. *J. Chem. Ed.* **1999**, *9*, 1260.

[S5] Melhuish, W. H. *J. Phys. Chem.***1961**, *65*, 229.

[S6] Jones II, G.; Jackson, W. R.; Choi, C.; Bergmark, W. R. *J. Phys. Chem.* **1985**, *89*, 294.

[S7] Lakowicz, J. R. Principles of Fluorescence Spectroscopy; Kluwer Academic/Plenum: New York, 1999; 2nd Ed.

[S8] Arbeloa, F. L.; Ojeda, P. R.; Arbeloa, I. L. *J. Luminescence* 2nd ed., **1989**, *44*, 105.

(5) X-ray structure of 25 and ^{1}H-NMR spectra of final compounds

Solid state structure of **25** with atom numbering:

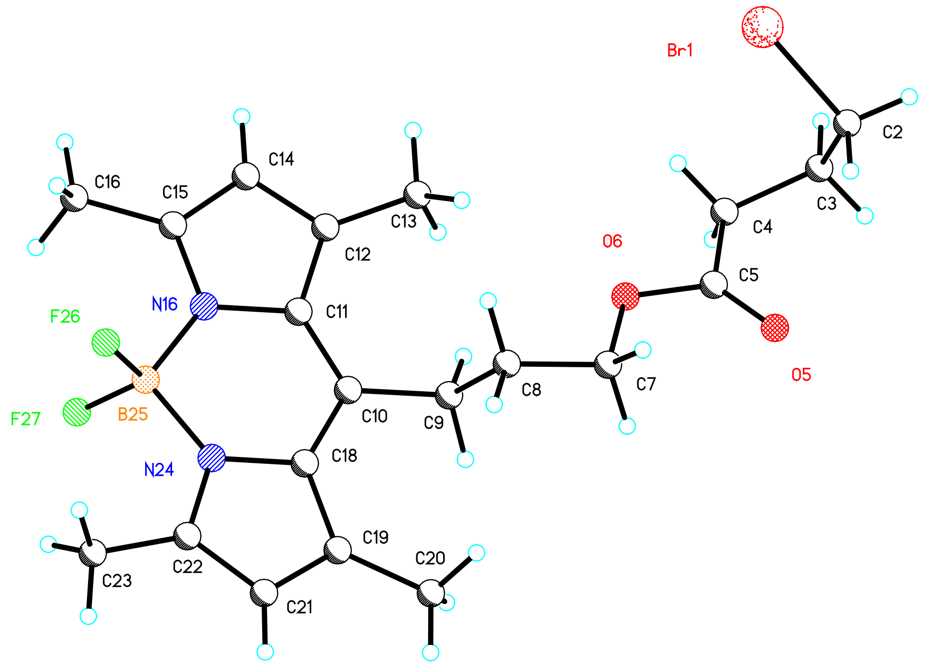

^{1}H-NMR spectrum of **14**:

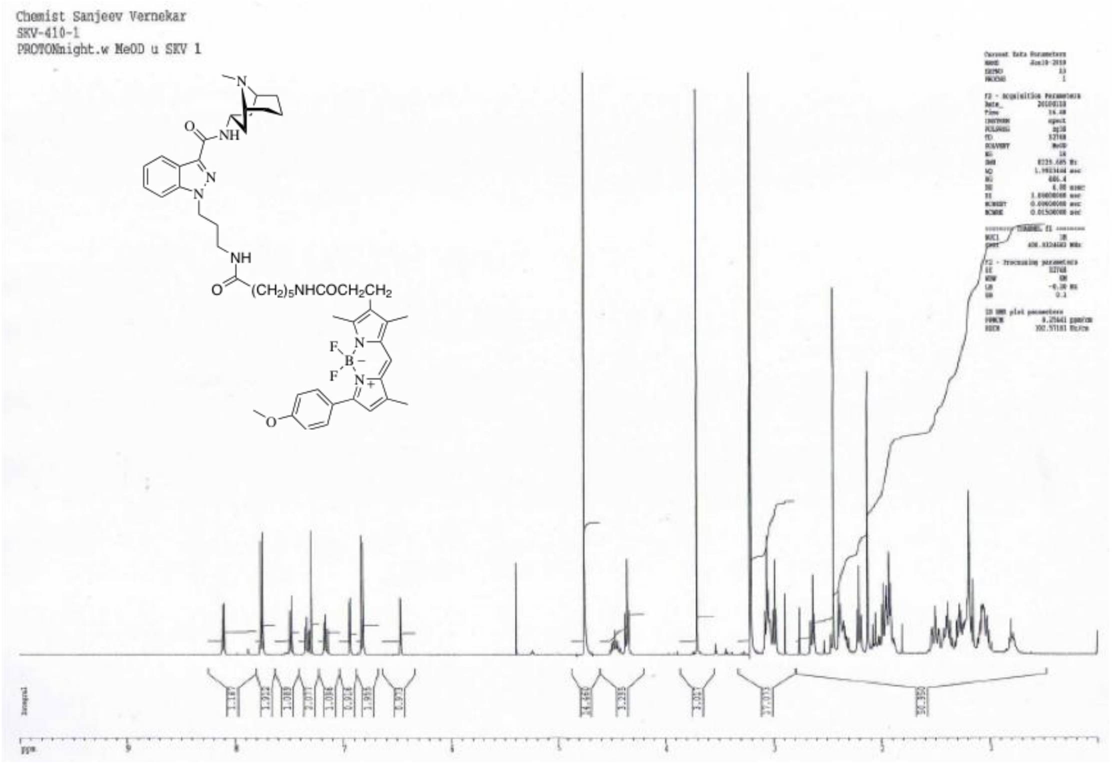

^{1}H-NMR spectrum of **15**:

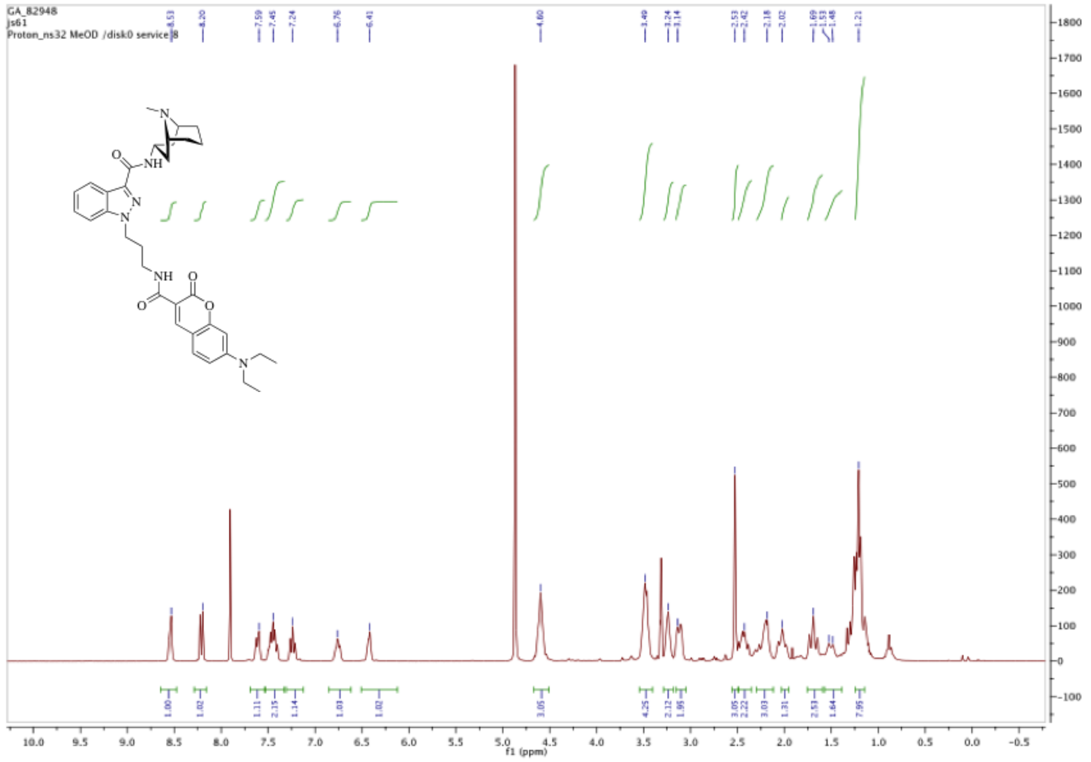

^{1}H-NMR spectrum of **16**:

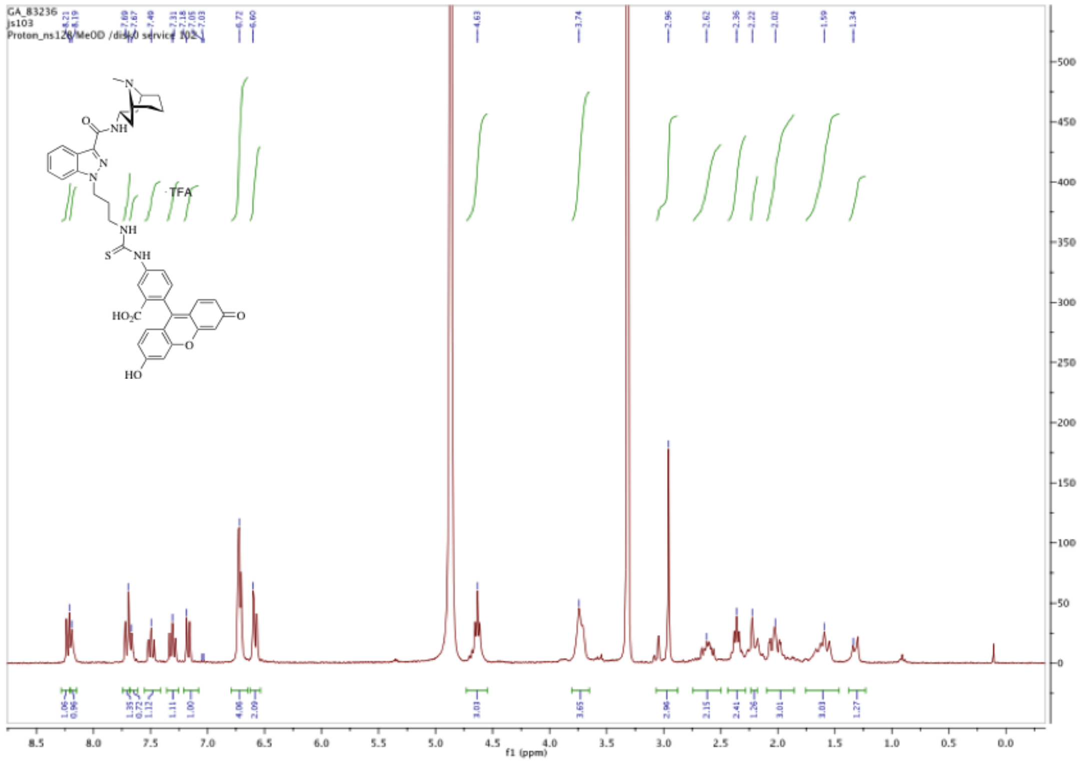

^{1}H-NMR spectrum of **17**:

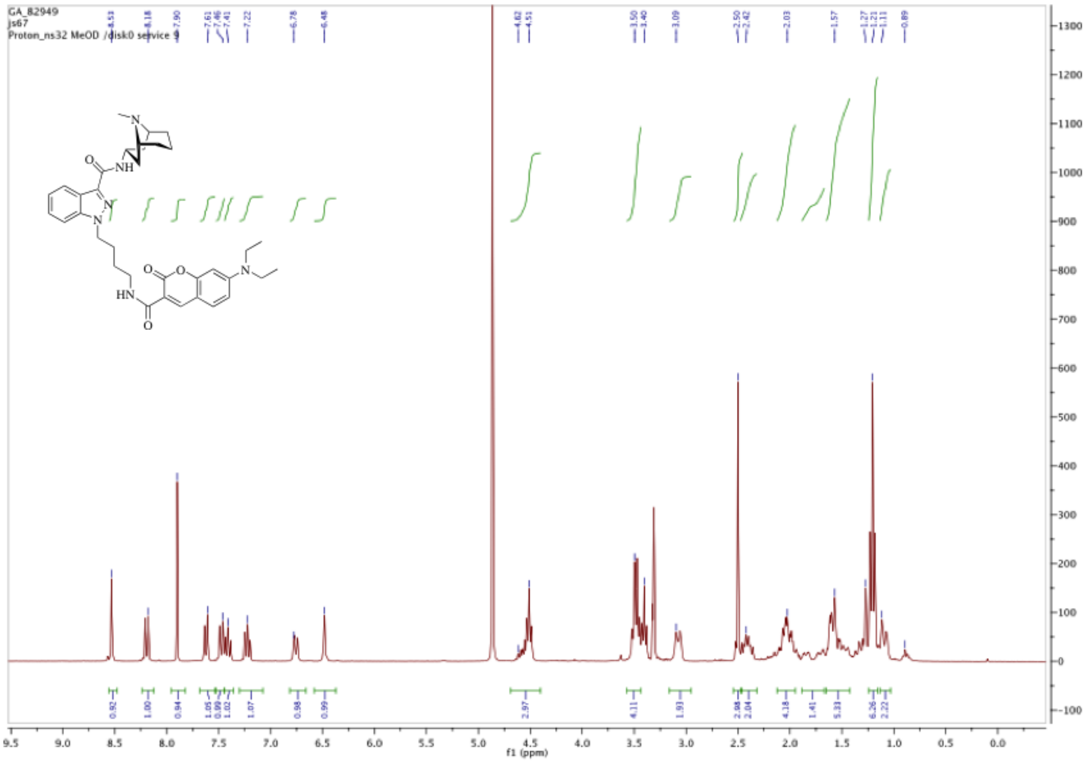

^{1}H-NMR spectrum of **18**:

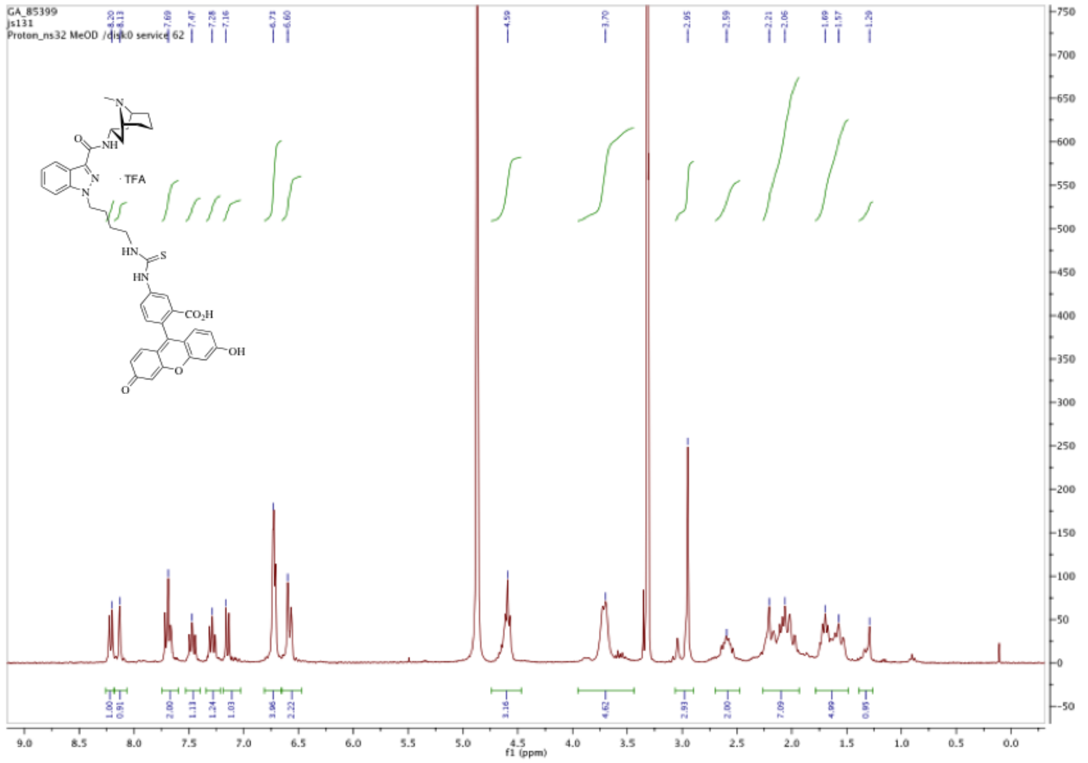

^{1}H-NMR spectrum of **19**:

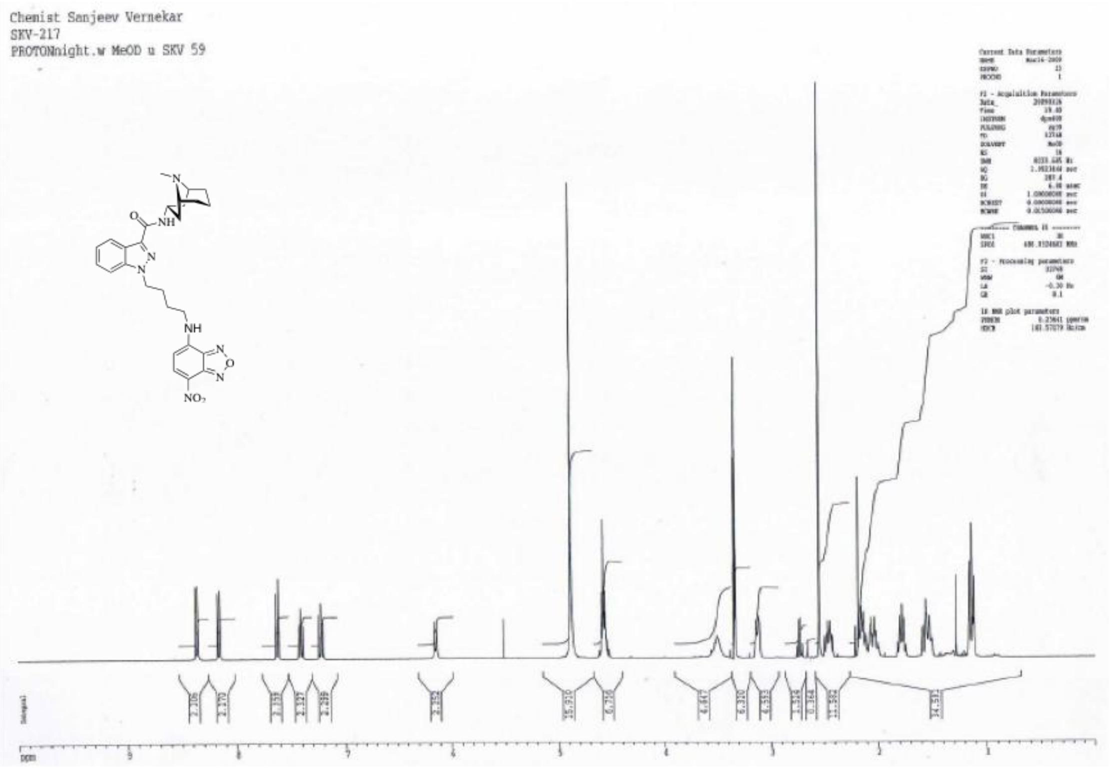

^{1}H-NMR spectrum of **20**:

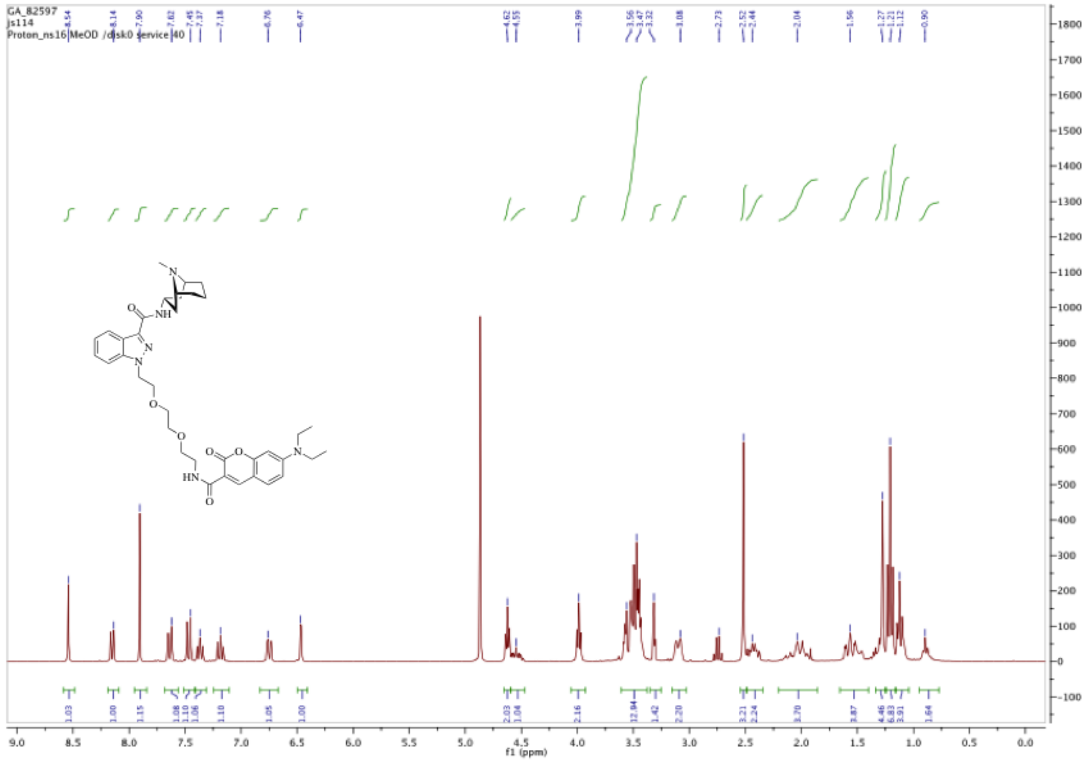

^{1}H-NMR spectrum of **21**:

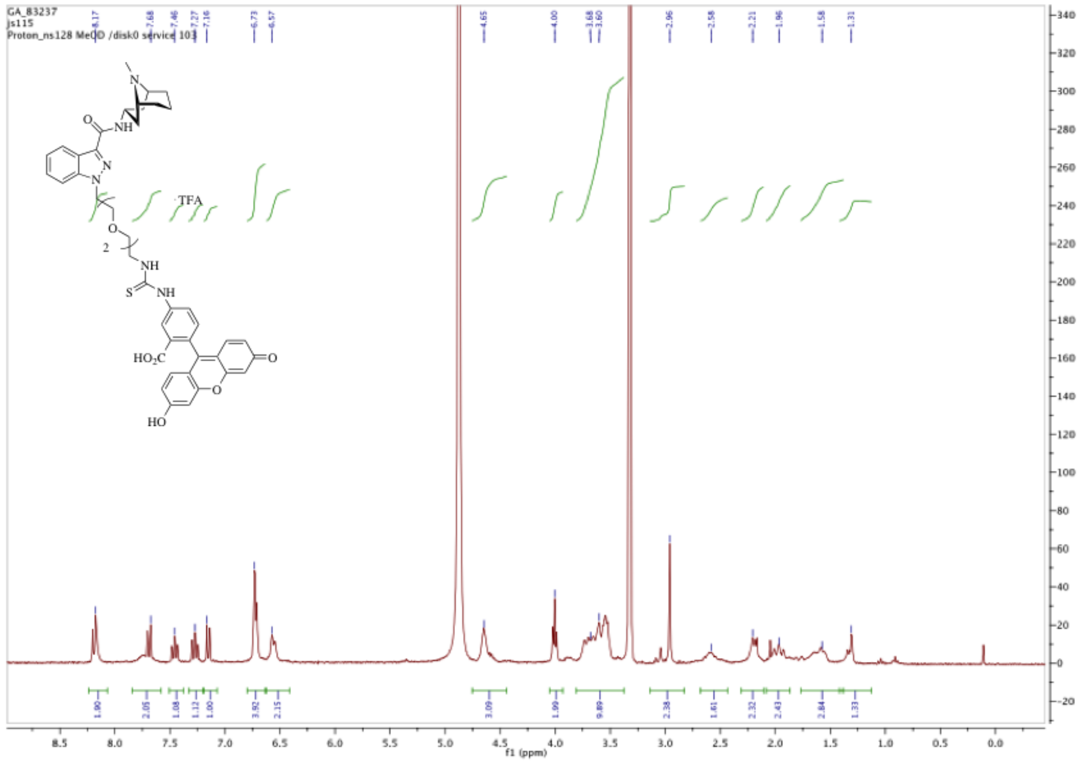

^{1}H-NMR spectrum of **22**:

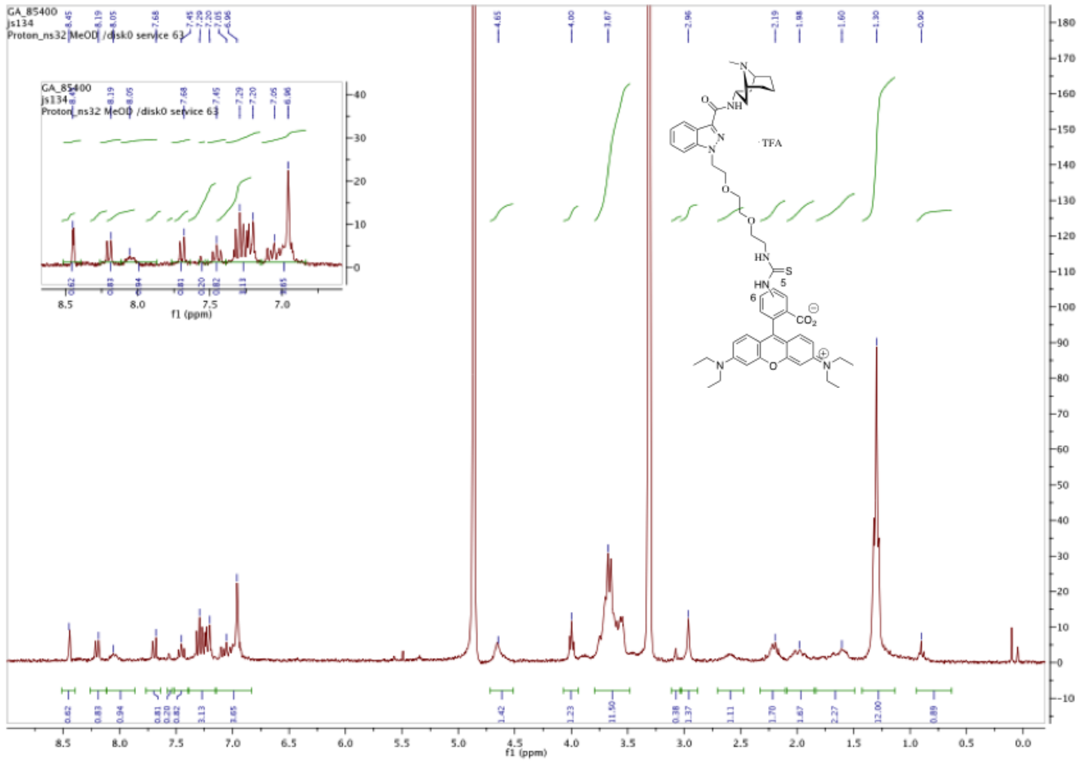

^{1}H-NMR spectrum of **26**:

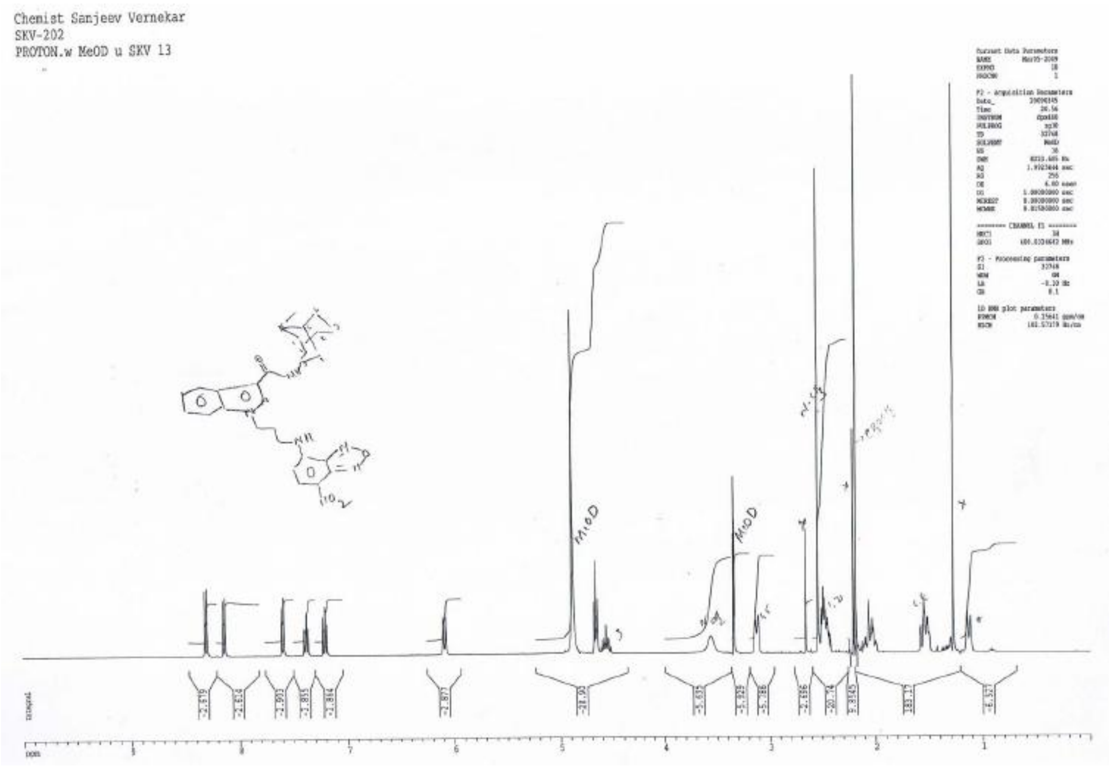

^{1}H-NMR spectrum of **27**:

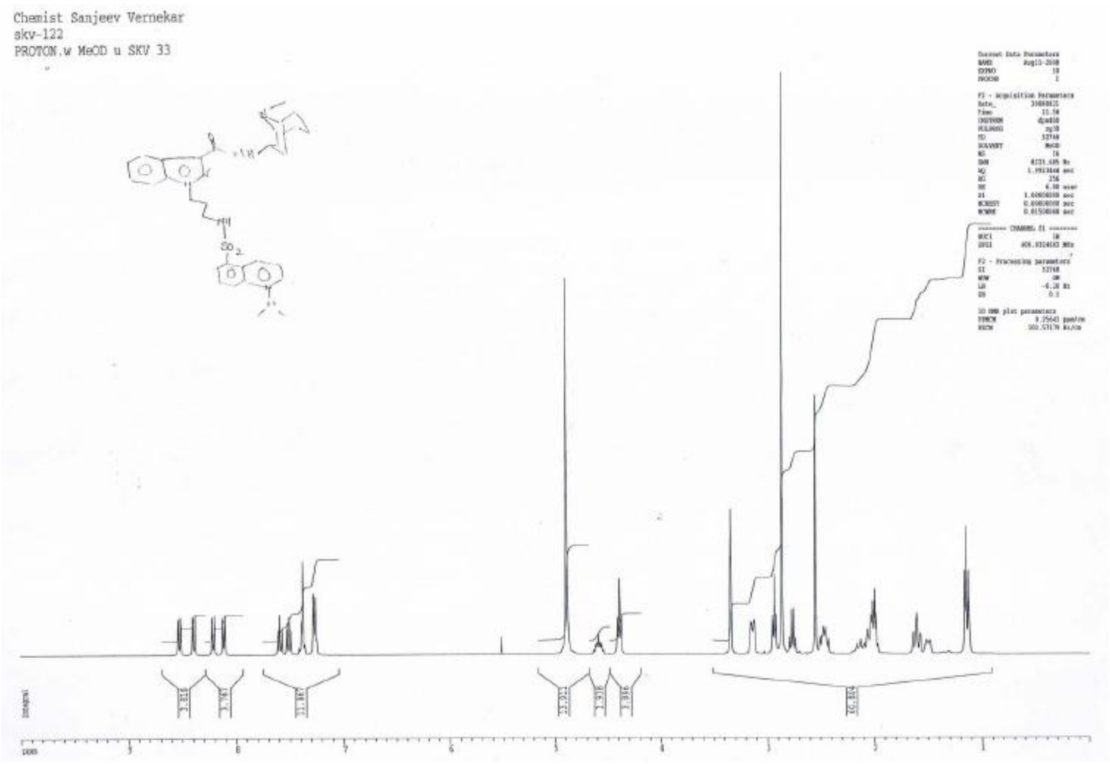

^{1}H-NMR spectrum of **28**:

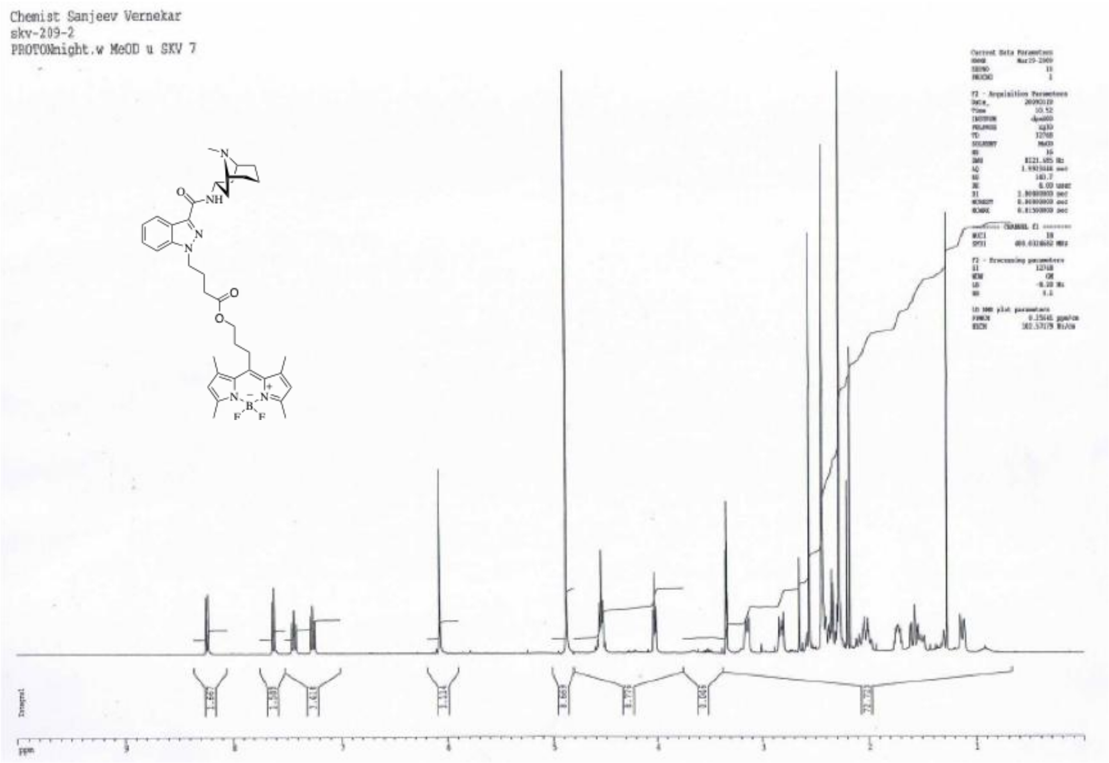

^{1}H-NMR spectrum of **36**:

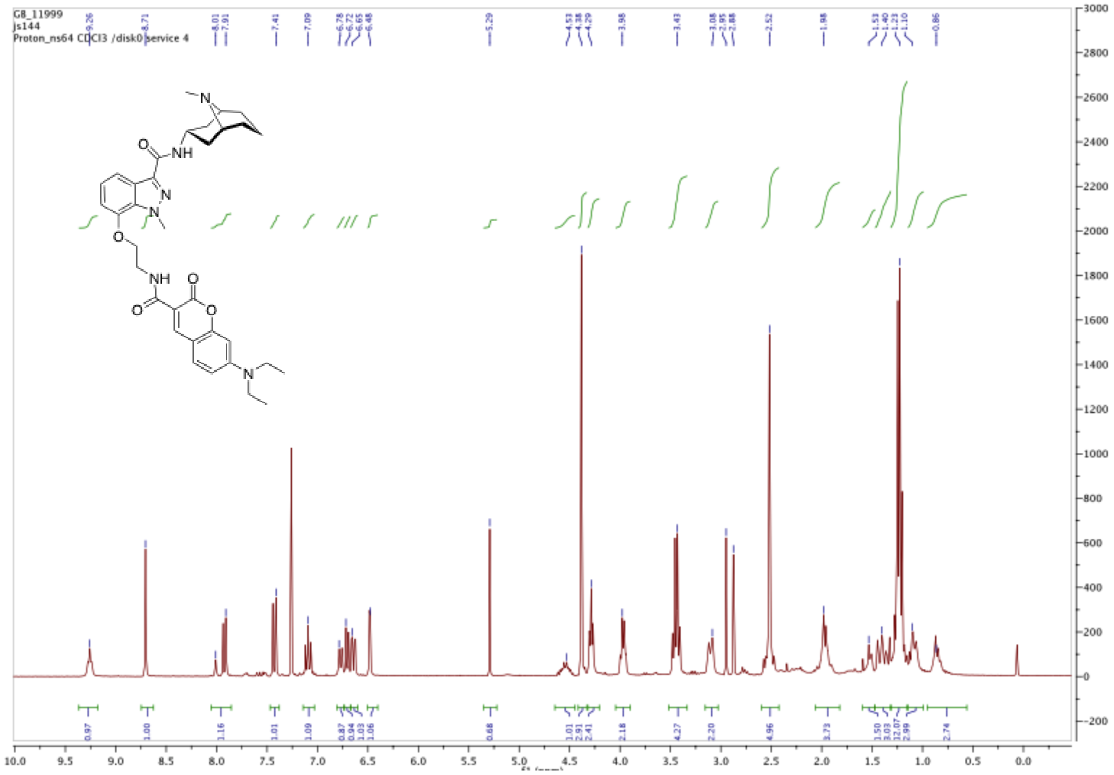

^{1}H-NMR spectrum of **37**:

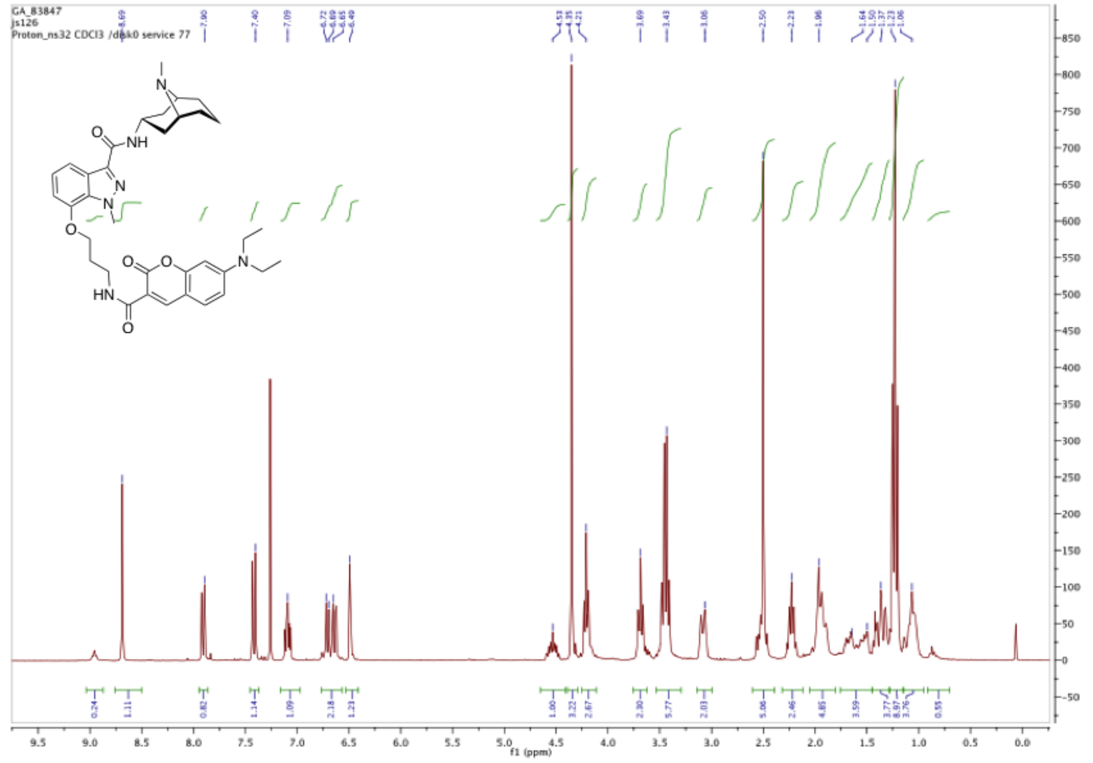

^{1}H-NMR spectrum of **38**:

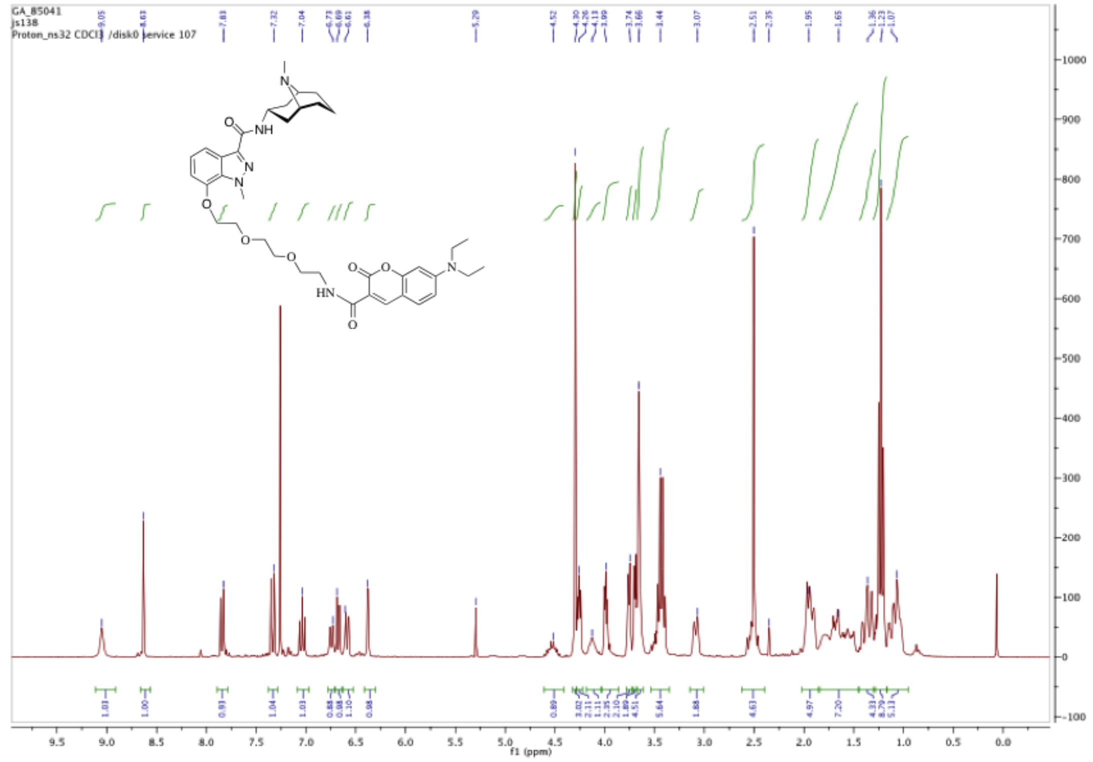

^{1}H-NMR spectrum of **39**:

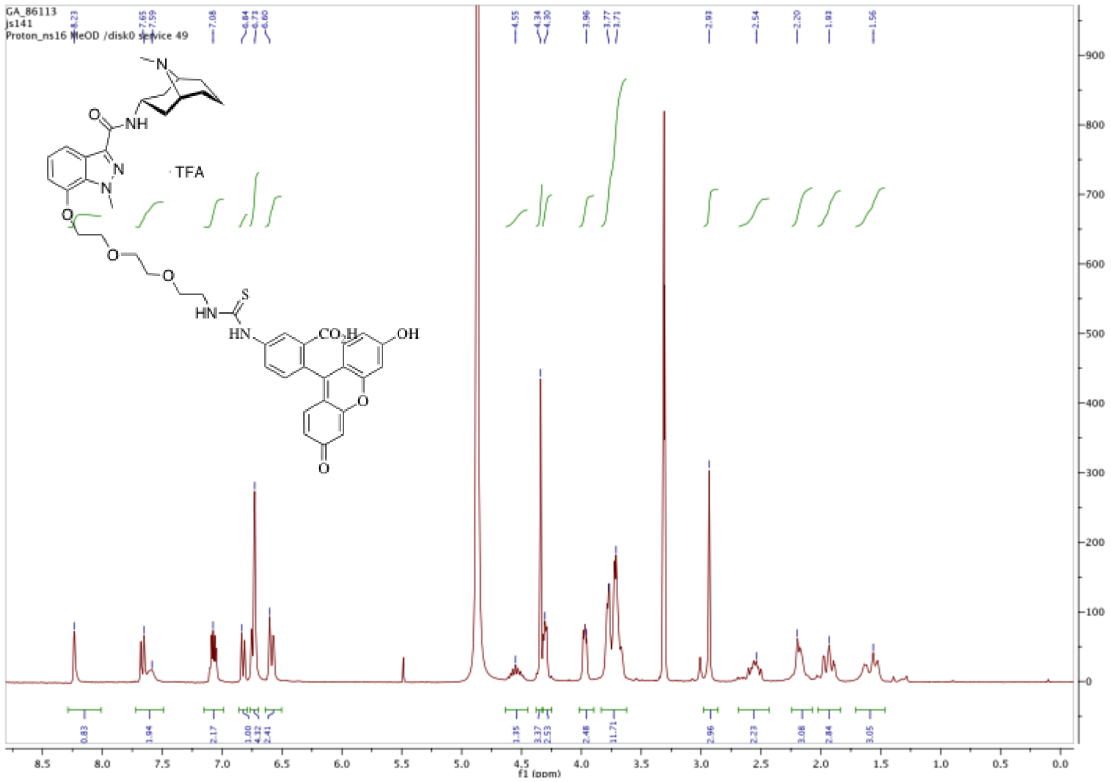

Supplement: Supplementary data — Synthesis details and spectral data for all compounds, crystal structure of 25, HPLC purity assessment for target compounds and experimental details for photophysical measurements. [file mmc1.pdf]
